# Cell-Based Double-Screening Method to Identify a Reliable Candidate for Osteogenesis-Targeting Compounds

**DOI:** 10.3390/biomedicines10020426

**Published:** 2022-02-11

**Authors:** Sho Fukuyasu, Hiroki Kayashima, Akihito Moribayashi, Shu Matsuoka, Atsuhiro Nagasaki, Hiroko Okawa, Hirofumi Yatani, Makio Saeki, Hiroshi Egusa

**Affiliations:** 1 Department of Fixed Prosthodontics, Osaka University Graduate School of Dentistry, 1-8 Yamadaoka, Osaka 565-0871, Japan; h-kaya@dent.osaka-u.ac.jp (H.K.); amoriba@dent.osaka-u.ac.jp (A.M.); matsuoka.shu.dent@osaka-u.ac.jp (S.M.); yatani@dent.osaka-u.ac.jp (H.Y.); 2 Fukuyasu Dental Clinic, 21-2 Otoshi-cho, Osaka 572-0048, Japan; 3 Division of Molecular and Regenerative Prosthodontics, Tohoku University Graduate School of Dentistry, 4-1 Seiryo-cho, Aoba-ku, Sendai 980-8575, Japan; atsuhiro.nagasaki.a5@tohoku.ac.jp (A.N.); hiroko.okawa.d3@tohoku.ac.jp (H.O.); 4 Division of Dental Pharmacology, Niigata University Graduate School of Medical and Dental Sciences, 2-5274 Gakkochodori, Niigata 951-8514, Japan; msaeki@dent.niigata-u.ac.jp; 5 Center for Advanced Stem Cell and Regenerative Research, Tohoku University Graduate School of Dentistry, Sendai 980-8575, Japan

**Keywords:** bone defect, compound, library screening, osteogenesis, tissue engineering

## Abstract

Small-molecule compounds strongly affecting osteogenesis can form the basis of effective therapeutic strategies in bone regenerative medicine. A cell-based high-throughput screening system might be a powerful tool for identifying osteoblast-targeting candidates; however, this approach is generally limited with using only one molecule as a cell-based sensor that does not always reflect the activation of the osteogenic phenotype. In the present study, we used the MC3T3-E1 cell line stably transfected with the green fluorescent protein (GFP) reporter gene driven by a fragment of type I collagen promoter (Col-1a1GFP-MC3T3-E1) to evaluate a double-screening system to identify osteogenic inducible compounds using a combination of a cell-based reporter assay and detection of alkaline phosphatase (ALP) activity. Col-1a1GFP-MC3T3-E1 cells were cultured in an osteogenic induction medium after library screening of 1280 pharmacologically active compounds (Lopack^1280^). After 7 days, GFP fluorescence was measured using a microplate reader. After 14 days of osteogenic induction, the cells were stained with ALP. Library screening using the Col-1a1/GFP reporter and ALP staining assay detected three candidates with significant osteogenic induction ability. Furthermore, leflunomide, one of the three detected candidates, significantly promoted new bone formation in vivo. Therefore, this double-screening method could identify candidates for osteogenesis-targeting compounds more reliably than conventional methods.

## 1. Introduction

Tissue engineering is a regenerative approach for tissue regeneration or replacement of damaged tissues using cells, scaffolds, and bioactive factors [1]. Several surgical strategies based on the tissue engineering concept have been used in bone tissue engineering, including autogenous bone grafting and stem cell transplantation, scaffolds, and growth factors [2]. However, unavoidable operative stress and a lack of cost-effectiveness are potential issues with using these surgical and stem cell-based therapies [3].

Ideal and standard regenerative and antiresorptive treatments for bone diseases can be achieved by developing effective, safe, and low-cost drugs and biomaterials [4]. Bones are constantly remodeled through the coupling of bone resorption and formation by osteoclasts and osteoblasts, respectively. Several growth factors, such as platelet-derived growth factors (PDGFs) [5], bone morphogenetic protein 2 (BMP2) [6], and basic fibroblast growth factor (b-FGF) [7], have been used to target osteoblasts to enhance bone regeneration. Although these growth factor proteins are promising, there are associated drawbacks, including comparatively high cost, supraphysiological dose, immunogenic potential [8], instability in vivo, and difficulty with sterilization [9].

Small-molecule compounds have various biological functions, serving as cell signaling molecules, molecular biology tools, and drugs in medicine [10]. Small-molecule compounds are advantageous because of their ease of synthesis and handling, and their inexpensiveness compared to growth factor proteins. The additional advantages of small-molecule compounds are their ability to penetrate the cell membrane, facilitating quick and reversible activation and inhibition of multiple specific targets and exhibiting synergistic effects on targeting osteoblasts to enhance osteogenesis [4]. Accordingly, identifying osteogenic inducible small molecules is a promising strategy for effective bone regenerative therapy.

High-throughput screening of small-molecule compound libraries is a powerful tool for drug discovery and biological mechanism elucidation [11]. There are various types of small-molecule compound libraries that contain functionally known or unknown compounds, synthetic or natural compounds and their derivatives, and therapeutic drugs. One of the most commonly used approaches for drug screening is a cell-based assay using genetically modified cells to regulate the expression of a reporter under the control of the target gene promoter [12]. However, one limitation of existing cell-based screening assays is that activating only one molecule as a cell-based sensor does not always reflect the activation of the osteogenic phenotype. During osteoblastic differentiation, alkaline phosphatase (ALP) activity is an early marker easily detected using conventional ALP staining methods.

Given the above, we hypothesized that combining a cell-based reporter assay and detecting ALP activity using a simple staining method would be a powerful screening system for identifying osteogenic inducible compounds. The present study aimed to evaluate the utility of a screening system that detects the expression of two osteogenic markers, type I collagen and ALP, to identify osteogenesis-targeting compounds.

## 2. Materials and Methods

### 2.1. Ethical Considerations

All animal experiments in the present study strictly followed a protocol approved by the Institutional Animal Care and Use Committee of the Osaka University Graduate School of Dentistry (approval number: 19-054).

### 2.2. Cell Cultures

Pre-osteoblastic MC3T3-E1 cell lines were purchased from the RIKEN Cell Bank (RCB1126; Ibaragi, Japan). MC3T3-E1 cell lines stably transfected with the green fluorescent protein (GFP) reporter gene driven by a 2.3 kb fragment of rat type I collagen promoter (Col-1a1GFP-MC3T3-E1) [12] were graciously supplied by Drs. Hironori Hojo and Ung-il Chung (University of Tokyo, Japan). MC3T3-E1 cells and Col-1a1GFP-MC3T3-E1 cells were cultured in MC3T3-E1 growth medium, which consisted of α-MEM (Nacalai Tesque, Kyoto, Japan) containing 10% fetal bovine serum (FBS; Equitech-Bio, Kerrville, TX, USA), 100 units/mL penicillin, 100 µg/mL streptomycin, and 250 ng/mL amphotericin B (Thermo Fisher Scientific, Waltham, MA, USA). For osteogenic induction, MC3T3-E1 cells and Col-1a1GFP-MC3T3-E1 cells were cultured in Dulbecco’s Modified Eagle’s Medium (Sigma, St. Louis, MO, USA) supplemented with 10% FBS, 0.1 μM dexamethasone (Sigma), 10 mM β-glycerophosphate (Sigma), 50 μM ascorbate-2-phosphate (Sigma), 100 units/mL penicillin, 100 µg/mL streptomycin, and 250 ng/mL amphotericin B.

Clonal mouse mesenchymal stem cells (mMSCs), which were established from mouse femur bone marrow [13], are multipotent, as demonstrated by their ability to differentiate specifically into osteoblasts, adipocytes, chondrocytes, and myoblast lineages. mMSCs were cultured in a growth medium consisting of α-MEM, 15% FBS (Thermo Fisher Scientific), 100 units/mL penicillin, 100 μg/mL streptomycin, and 250 ng/mL amphotericin B. Rat bone marrow-derived MSCs (rMSCs) were isolated from 8-week-old Sprague-Dawley rats as described previously [14]. For osteogenic induction, these MSCs were cultured in an osteogenic induction medium consisting of α-MEM, 15% FBS (Thermo Fisher Scientific), 0.1 µM dexamethasone, 10 mM β-glycerophosphate, 50 µM ascorbate-2-phosphate, 100 units/mL penicillin, 100 µg/mL streptomycin, and 250 ng/mL amphotericin B [15].

### 2.3. Small-Molecule Compounds

Prior to library screening, the reliability of the screening method was confirmed using known osteogenic inducible bioactive factors, harmine (Biomol, Plymouth Meeting, PA, USA) [16], phenamil (Sigma) [17,18], resveratrol (Sigma) [19,20,21,22], and recombinant human BMP2 [23,24,25] (Peprotech, London, UK) as positive controls. After library screening, the candidates for osteogenesis targeting, leflunomide (Lef), 1-(5-isoquinolinylsulfonil)-3-metylpiperazine dihydrochloride (1-5), and LFM-A13 (LFM), were purchased from Sigma, Santa Cruz Biotechnology (Dallas, TX, USA) and Calbiochem (Beeston Nottingham, UK), respectively, to confirm their abilities.

### 2.4. Double Detection of GFP and ALP Expressions

Col-1a1GFP-MC3T3-E1 cells were seeded into 96-well culture plates (black wall and clear bottom) for fluorescence-based assays at a density of 16,000 cells per well in MC3T3-E1 growth medium. The next day, the culture medium was exchanged for a fresh osteogenic induction medium containing 1 or 10 µM of osteogenic inducible small molecules (harmine, phenamil, and resveratrol) or 100 ng/mL BMP2. The medium was changed every two days.

GFP fluorescence in each well was measured 7 days after induction using a fluorescence microplate reader (GloMax-Multi Detection System; Promega, Madison, WI, USA). After the measurement, the cells were cultured in an osteogenic induction medium for another 7 days. After 14 days of osteogenic induction, a standard ALP staining method [15] was used to detect ALP activity in each well. Colorimetric analysis of ALP activity was performed as described previously [26], by measuring the optical density at a wavelength of 405 nm.

After establishing the cell-based double-screening system, 1280 pharmacologically active compounds (10 µM) from a small-molecule library (Lopack^1280^; Sigma) [27] were used in the screening assay. A list of the compounds in the Lopack^1280^ library is shown in Supplementary Table S1.

### 2.5. Cytotoxicity and Cell Proliferation Assays

MC3T3-E1 cells were seeded into 96-well tissue culture plates (1000 cells per well) and maintained in a growth medium for 24 h. The medium was replaced with a growth medium containing 0, 1, 10, 25, and 50 µM candidate compounds (Lef, 1-5, and LFM). The cells were then cultured for 5 and 6 days for the CytoTox-Glo luminescent cytotoxicity assay (Promega) [10] and WST-1 cell counting assay (Dojindo Laboratories, Kumamoto, Japan) [28] to evaluate cytotoxicity and cell proliferation, respectively.

### 2.6. Reverse Transcription Polymerase Chain Reaction (RT-PCR) Analyses

Total RNA was isolated using the RNeasy Mini Kit (Qiagen, Hilden, Germany). After DNase treatment (Thermo Fisher Scientific), cDNA was synthesized from 1 µg of total RNA using SuperScript III reverse transcription (Thermo Fisher Scientific). Real-time quantitative RT-PCR analysis was performed using Thunderbird SYBR qPCR Mix (Toyobo, Osaka, Japan) on an ABI PRISM 7900 Sequence Detection System (Thermo Fisher Scientific). The mRNA expression of osteogenic marker genes (*Osterix*, *Collagen 1a1*, *Runx2*, and *Osteocalcin*) was determined using glyceraldehyde-3-phosphate dehydrogenase (*Gapdh*) as internal control. The primer pairs and sequences are shown in Supplementary Table S2.

### 2.7. Evaluation of the Candidate Molecules Using MSCs

Candidates of osteogenic inducible compounds (10 µM) by the library screening were added to mMSCs and rMSCs in the osteogenic induction medium, and cells were cultured for 21 days. To evaluate the effects of the candidates on the osteogenic differentiation of these cells, ALP/von Kossa staining was performed as described previously [15].

### 2.8. Lef Injection into Rat Calvarial Bone Defects

Eight-week-old male Sprague-Dawley rats were anesthetized, and a circular defect of 5 mm in diameter was formed at the calvaria [28]. Lef injection was performed as previously described [29,30] with minor modifications. Briefly, after the defect was formed, collagen graft material (Terudermis, Olympus Terumo Biomaterials, Tokyo, Japan) containing 0.5 or 5 µg of Lef was transplanted into the bone defects. Total 3 or 30 µg dosage of Lef was applied to each defect site dividing into 5 injections (0.5 or 5 µg for each injection) every three or four days. The same volume of saline was applied to the defect in the control group. Three weeks after the operation, calvariae were extracted for histological (hematoxylin and eosin (H&E) and TRAP staining) [28] and three-dimensional micro-computed tomography (CT) (R_mCT2; RIGAKU, Tokyo, Japan) analyses [31] of new bone formation. Bone density inside the defect was measured using the bone tissue analysis software program (TRI/3D-BON; RATOC System Engineering, Tokyo, Japan). For histological and micro-CT analyses, 10 and 7 samples, respectively, from different mice were used.

### 2.9. Statistical Analysis

One-way analysis of variance (ANOVA) with Dunnett’s post hoc test was used to evaluate the statistical significance of the results. Statistical significance was defined as *p* < 0.05.

## 3. Results

### 3.1. Verification of ALP and GFP Expression in Col-1a1GFP-MC3T3-E1 Cells

**Figure 1:**
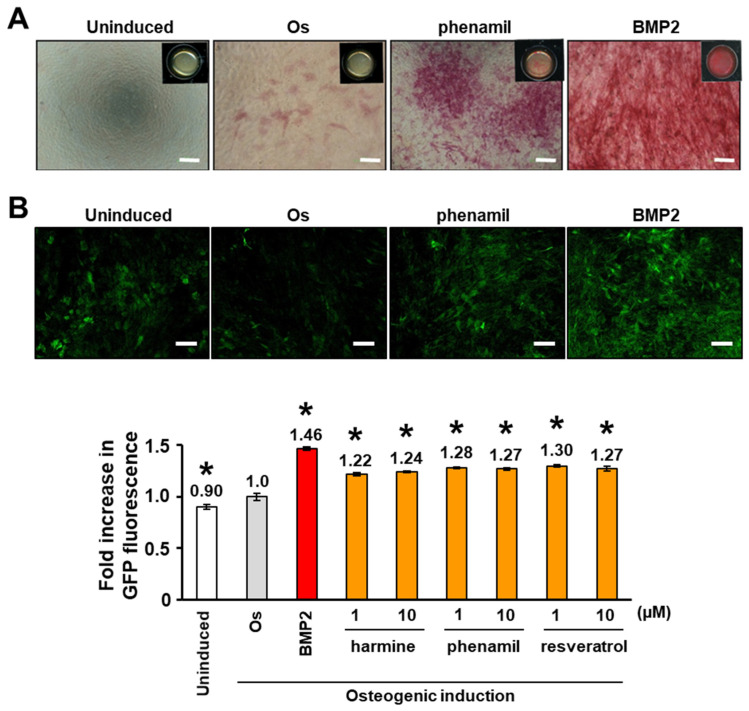
Verification of ALP and GFP expression of Col-1a1GFP-MC3T3E1 cells. Pre-osteoblastic Col1a1GFP-MC3T3-E1 cells, stably transfected with the GFP reporter gene driven by a fragment of rat type I collagen promoter, were cultured in 96-well culture plates for 14 days in the growth medium (Uninduced) or osteogenic induction medium (Os) in the presence or absence of 1 or 10 µM of osteogenic inducible compounds (harmine, phenamil, and resveratrol) or BMP2 (100 ng/mL). (**A**) Col-1a1GFP-MC3T3-E1 cells cultured under Os conditions with phenamil (1 µM) or BMP2 strongly expressed ALP on day 14. Representative image from three independent experiments are shown. Bars: 100 µm. (**B**) GFP fluorescence of Col-1a1GFP-MC3T3-E1 cell culture under each condition was measured on day 7. Representative fluorescence images from three independent experiments are shown. Values shown are means ± standard errors of the means (*n*-fold increase) in the GFP fluorescence intensity compared to that under Os conditions from three independent experiments (*n* = 3). Significant differences (* *p* < 0.01: ANOVA with Dunnett’s correction for multiple comparisons) were evaluated with respect to the Os condition values.

To verify the enhanced ALP activity and GFP expression in Col-1a1GFP-MC3T3-E1 cells in response to osteogenic induction factors, the cells were cultured in an osteogenic induction medium in the presence of phenamil and BMP2 for 14 days. Col-1a1GFP-MC3T3-E1 cells in the osteogenic induction medium showed slight ALP activity on day 14 (Figure 1A). In contrast, robust ALP activity in Col-1a1GFP-MC3T3-E1 cells was confirmed in osteogenic induction medium containing phenamil (1 µM) and BMP2 (100 ng/mL). ALP activity was not detected in uninduced cells cultured in the growth medium.

Seven days after osteogenic induction, the fluorescence measurement results showed that Col-1a1GFP-MC3T3-E1 cells emitted significantly more GFP fluorescence intensity than uninduced cells (*p* < 0.01) (Figure 1B). Both 1 and 10 µM of osteogenic inducible small molecules, including harmine, phenamil, and resveratrol, and 100 ng/mL BMP2, significantly enhanced the expression of GFP fluorescence in Col-1a1GFP-MC3T3E1 cells cultured in osteogenic induction medium (*p* < 0.01) (Figure 1B). The fold increase in GFP intensity for harmine, phenamil, and resveratrol at 10 µM exceeded 1.24.

### 3.2. Library Screening Using the Double-Screening Method

**Figure 2:**
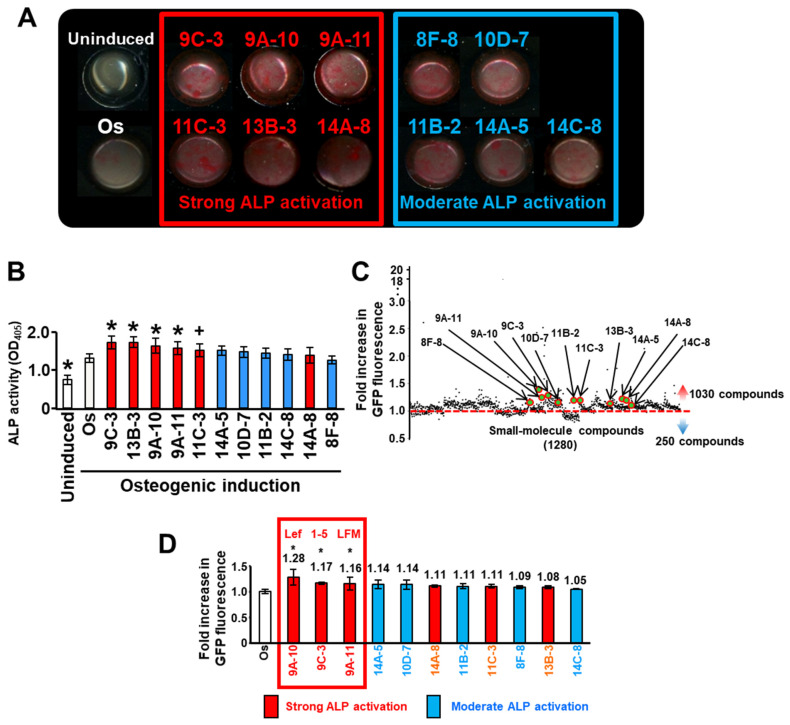
Library screening of Lopack^1280^ using the cell-based double-screening method. The Lopack^1280^ library compounds in a 96-well format were added to Col1a1GFP-MC3T3-E1 cells in osteogenic induction medium (Os) at 10 µM concentration. (**A**) ALP staining on day 14 showed that six compounds (9C-3, 9A-10, 9A-11, 11C-3, 13B-3, and 14A-8) strongly activated ALP in Col-1a1GFP-MC3T3-E1 cells, and five compounds (8F-8, 10D-7, 11B-2, 14A-5, and 14C-8) moderately activated ALP. Representative image from three independent experiments are shown. A description of each compound is shown in Table 1. (**B**) Colorimetric analysis of ALP activity assay using MC3T3-E-1 cells in response to the 11 compounds. The data represent the mean values ± standard deviation (*n* = 10). Significant differences (^+^ *p* < 0.05, * *p* < 0.01: ANOVA with Dunnett’s correction for multiple comparisons) were evaluated in comparison to the control (Os) values. (**C**) GFP fluorescence of Col-1a1GFP-MC3T3-E1 cells in each well was measured after 7 days of treatment with each compound. Each dot represents each small-molecule compound with the mean values (*n* = 3) of the fold increase with respect to the mean value under Os conditions from three independent experiments. (**D**) GFP fluorescence intensity on day 7 for the six strong ALP activators (red bars) and five moderate ALP activators (blue bars). Values given are means ± standard errors of the means (*n*-fold increase) in the GFP fluorescence intensity compared to that under Os conditions from three independent experiments (*n* = 3). Significant differences (* *p* < 0.01: ANOVA with Dunnett’s correction for multiple comparisons) were evaluated with respect to the Os condition values. Three candidate compounds, leflunomide (Lef), and 1-(5-isoquinolinylsulfonil)-3-metylpiperazine dihydrochloride (1-5), LFM-A13 (LFM) were identified.

ALP staining showed that six compounds (Lef, LFM, 1-5, H-8 dihydrochrloride [H-8], putrescine dihydrochloride, and spermide trihydrochloride) strongly stained Col-1a1GFP-MC3T3-E1 cells red, and five compounds (Ro 41-0960, MRS 2179, L-alpha-Methyl-p-tyrosine, L-165,041, and SKF 83959 hydrobromide) moderately stained those cells red (Table 1 and Figure 2A). Colorimetric analysis confirmed that these 11 compounds showed higher ALP activity in MC3T3-E1 cells than the control group (Figure 2B). Particularly, five (Lef, LFM, 1-5, H-8, and putrescine dihydrochloride) of the six strongly stained compounds in Col-1a1GFP-MC3T3-E1 cells significantly promoted ALP activity in MC3T3-E1 cells.

In contrast, the library screening of Lopack^1280^ by GFP fluorescence measurement on day 7 showed that 1030 compounds induced GFP expression in cells compared to the control conditions (in osteogenic induction medium without compounds) (Figure 2C). The fold increase in cellular GFP fluorescence intensity for these 11 ALP activator compounds ranged from 1.05 to 1.28 times higher than that in the control conditions (Figure 2D). Among these 11 ALP activator compounds, Lef, LFM, and 1-5 significantly increased the cellular GFP fluorescence intensity (1.28-, 1.17-, and 1.16-fold, respectively) compared to the control condition (*p* < 0.01) (Figure 2D).

### 3.3. Effects of Identified Candidate Compounds on Cytotoxicity and Cell Proliferation

A proliferation assay showed that 1 and 10 µM Lef, LFM, and 1-5 did not significantly affect the proliferation of MC3T3-E1 cells cultured for 6 days in the growth medium. In contrast, 25 and 50 µM of Lef and 1-5 significantly suppressed the proliferation of MC3T3-E1 cells at 6 days after treatment (Supplementary Figure S1A). Cytotoxicity assay showed that 1, 10, 25, and 50 µM of these candidate compounds did not significantly affect the survival of MC3T3-E1 cells after 5 days of culture under compound stimulation (Supplementary Figure S1B).

### 3.4. Effects of Identified Candidate Compounds on Osteogenesis In Vitro

**Figure 3:**
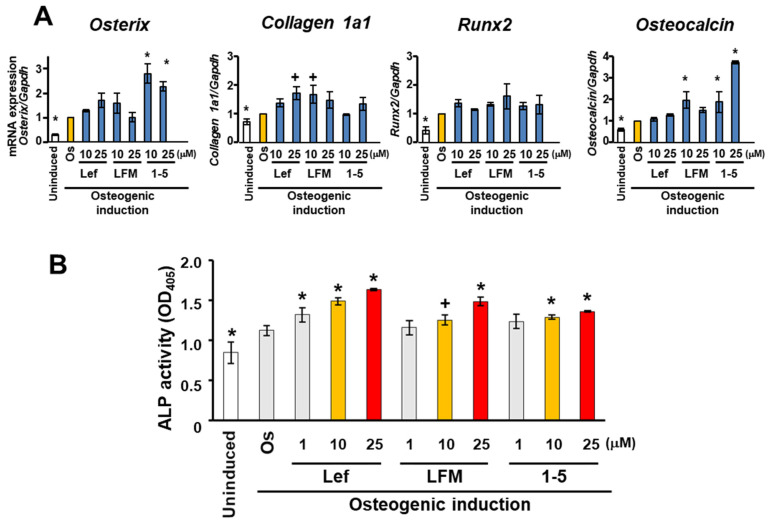
Effects of identified candidate compounds on the osteogenesis of MC3T3-E1 cells. MC3T3-E1 cells were cultured in a growth medium (Uninduced) or osteogenic induction medium (Os) with 1–25 μM leflunomide (Lef), LFM-A13 (LFM), and 1-(5-isoquinolinylsulfonil)-3-metylpiperazine dihydrochloride (1-5). (**A**) Expression of osteogenic marker genes, *Osterix, Collagen 1a1, Runx2, and Osteocalcin* was evaluated using a real-time RT-PCR analysis on day 10. *Gapdh* was used as the internal control. Experiments were performed in triplicate and repeated three times with similar results. Representative data from three independent experiments are shown (mean values ± standard deviation: *n* = 3). Significant differences (^+^ *p* < 0.05, * *p* < 0.01: ANOVA with Dunnett’s correction for multiple comparisons) were evaluated in comparison to the control (Os) values. (**B**) Colorimetric analysis of ALP activity assay using MC3T3-E1 cells on day 14. The data represent the mean values ± standard deviation (*n* = 4). Significant differences (^+^ *p* < 0.05, * *p* < 0.01: ANOVA with Dunnett’s correction for multiple comparisons) were evaluated in comparison to the control (Os) values.

At this stage, three candidate compounds (Lef, LFM, and 1-5) were selected for further studies to evaluate their osteogenic induction activity. Quantitative RT-PCR showed an increasing trend in the expression of osteogenic marker genes (*Osterix*, *Collagen 1a1*, *Runx2*, and *Osteocalcin*) in MC3T3-E1 cells 10 days after osteogenic induction in the presence of Lef, LFM, and 1-5 (Figure 3A). In particular, 25 µM Lef, 10 µM LFM, and 10–25 µM 1-5 significantly promoted the expression of *Collagen 1a1* (*p* < 0.05), *Collagen 1a1* (*p* < 0.05) and *Osteocalcin* (*p* < 0.01), and *Osterix* and *Osteocalcin* (*p* < 0.01), respectively. In addition, these candidate compounds significantly promoted ALP activity in MC3T3-E1 cells on day 14 after osteogenic induction in a concentration-dependent manner (*p* < 0.05) (Figure 3B).

**Figure 4:**
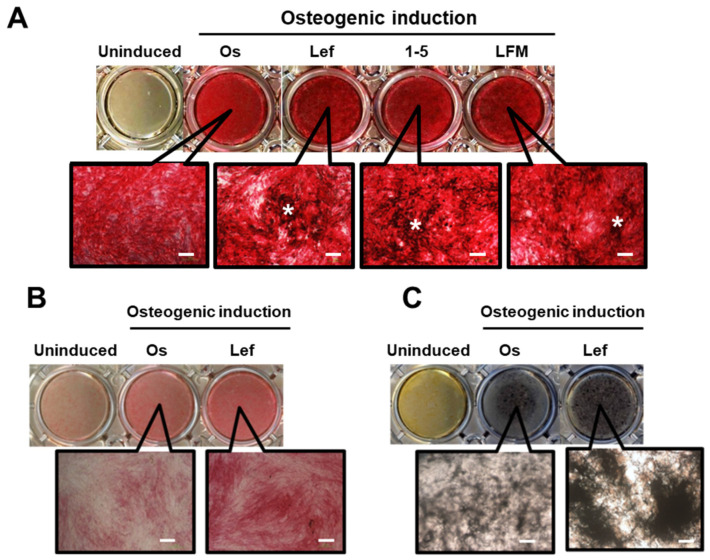
Effects of the identified candidate compounds on the osteogenesis of mouse and rat mesenchymal stem cells (mMSCs and rMSCs). mMSCs (**A**) and rMSCs (**B**,**C**) were cultured in a growth medium (Uninduced) or osteogenic induction medium (Os) with 10 μM leflunomide (Lef), LFM-A13 (LFM), and 1-(5-isoquinolinylsulfonil)-3-metylpiperazine dihydrochloride (1-5). (**A**) ALP/von Kossa staining was performed for mMSCs on day 14. Bars: 200 μM. Asterisks indicate von Kossa-positive nodule mineralization. (**B**) ALP or (**C** von Kossa staining was performed for rMSCs on days 14 and 21, respectively. Bars: 200 μM.

ALP/von Kossa staining confirmed a marked osteogenic induction in mMSCs, showing enhanced ALP activity and distinct extracellular matrix calcium deposition by 10 µM of Lef, LFM, and 1-5 on day 14 (Figure 4A). In addition, 10 µM Lef enhanced ALP activity and nodule mineralization in rMSCs (Figure 4B,C). LFM and 1-5 did not significantly enhance ALP activity and nodule mineralization of rMSCs; therefore, we selected Lef as a candidate for subsequent investigation in animal experiments.

### 3.5. Effects of Lef on Calvarial Bone Defect Regeneration

**Figure 5:**
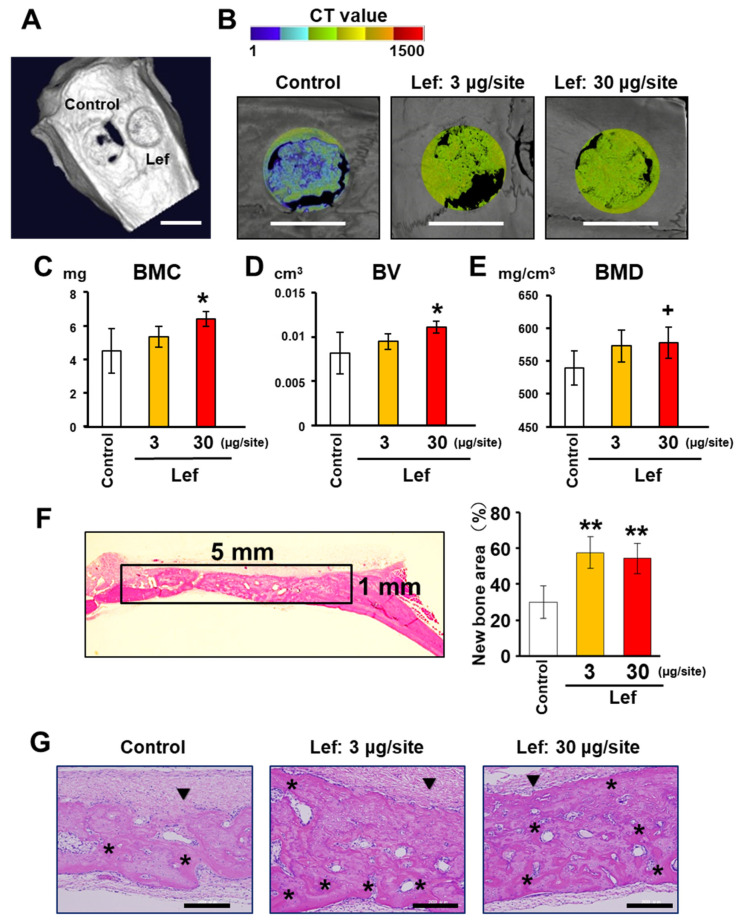
Micro-CT and histological analyses of the effects of leflunomide (Lef) on bone regeneration in the rat calvarial bone defect. After the defect (5 mm in diameter) had been formed, collagen graft material containing 0.5 or 5 µg of Lef was transplanted into the bone defects. Total 3 or 30 µg dosage of Lef was applied to each defect site dividing into 5 injections every three or four days. The same volume of saline was applied to the defect in the control group. At 3 weeks after the operation, micro-CT analysis was performed. (**A**) Representative micro-CT image of the extracted calvaria (Bar: 5 mm). (**B**) Micro-CT images demonstrated significant new bone formation in bone defects with Lef injection at 3 μM/site and 30 μM/site. Bars: 5 mm. (**C**–**E**) The bone mineral content (BMC) (**C**), bone volume (BV) (**D**) and bone mineral density (BMD) (**E**) at the defect area was measured. The data represent the mean values ± standard deviation (*n* = 7) from 7 independent experiments. Significant differences (⁺ *p* < 0.05, * *p* < 0.01: ANOVA with Dunnett’s correction for multiple comparisons) were evaluated in comparison to the control values. (**F**,**G**) Histological analysis of the effects of Lef on bone regeneration in the rat calvarial bone defect. At 3 weeks after the operation, calvariae were extracted for H&E staining. (**F**) Ratio of the newly formed bone area in the original calvarial defects (the surrounding area) was calculated. The data represent the mean values ± standard deviation (*n* = 10) from 10 independent experiments. Significant differences (** *p* < 0.001: ANOVA with Dunnett’s correction for multiple comparisons) were evaluated in comparison to the control values. (**G**) H&E staining showed that 3 and 30 μM/site Lef-injected areas achieved stronger repair of calvarial defects with new bone formation. Asterisks (*) indicate cement lines. ▼: Remaining collagen graft material. Bars: 200 µM.

Three weeks after the operation, Micro-CT images showed superior new bone formation in the bone defects injected with Lef at 3 and 30 μM/site compared to the control condition (Figure 5A,B). Micro-CT analysis demonstrated that the bone mineral content, volume, and density of bone regenerated by 30 μM/site were significantly higher than those in the control group (*p* < 0.05) (Figure 5C–E).

H&E staining showed that the areas of the regenerated bone at the sites injected with 3 and 30 μM/site Lef were significantly larger than those in the control condition (*p* < 0.01) (Figure 5F). Magnified images of 3 µM/site and 30 µM/site Lef-injected areas showed superior repair of calvarial defects with new bone formation compared to the control condition (Figure 5G). The newly formed bone in the Lef-injected areas showed clear cement lines, which is the histological profile of the remodeled compact bone [28]. The number of TRAP-positive multinucleated cells, which represent osteoclasts (Supplementary Figure S2A), in the bone defect regions of the Lef-injected groups was not significantly different from that of the control group (Supplementary Figure S2B).

## 4. Discussion

Chemical biology elucidates biological phenomena at the molecular level and is expected to be an essential step for drug discovery. A screening strategy based on a large-scale compound library requires more efficient and accurate high-throughput screening systems. To date, several cell-based screening systems have been reported [11], which detect cell proliferation [32], cytotoxicity [33], and differentiation [34]. Most library screening systems for osteoblasts have used the ALP activity index of MSCs and MC3T3 cells [35,36,37,38]. ALP activity is a marker of early osteogenic differentiation [39,40] and can be easily detected using staining. However, ALP staining is necessary to fix cells, making it difficult to evaluate other osteogenic differentiation markers using the same cell culture. Therefore, many studies have described using cell proliferation assays to double-screen indices not related to differentiation [37,38].

A screening system with an index set for several differentiation markers, rather than just one, is ideal for detecting compounds affecting osteogenic differentiation [41]. In addition, the detection methods for cell response must be simple and the detected data are quantitative. In the present study, we applied our new double-screening system using ALP staining and pre-osteoblastic Col-1a1GFP-MC3T3-E1 cell lines stably transfected with the GFP reporter gene driven using a fragment of the type I collagen promoter [12]. Type I collagen is an osteogenic marker; therefore, the cells in the present study robustly emitted GFP fluorescence in response to osteogenic activators such as BMP2 [23,24,25], harmine [16], phenamil [17,18], and resveratrol [19,20,21,22]. However, one limitation of this GFP fluorescence-based screening assay is that detecting only type I collagen expression does not always reflect the activation of the osteogenic phenotype. In addition, proteasome inhibitors, such as MG-132, inhibit the degradation of GFP, resulting in the detection of pseudo-GFP expression [42]. The GFP fluorescence detected in this system was not very high and that of BMP2 was 1.46-fold higher than that in osteogenic-induced cells. Additionally, compound AC-93253 iodide showed the greatest increase in GFP fluorescence (18.8-fold); however, it did not activate ALP in Col-1a1GFP-MC3T3-E1 cells. Therefore, we applied the detection of ALP, another early osteogenic marker, in combination with a GFP fluorescence-based screening assay.

By targeting two early osteogenic markers in the same pre-osteoblast culture, we evaluated the Lopack^1280^ library of pharmacologically active small molecules. Such small-molecule screening assays have attracted substantial attention in recent years as drug discovery tools and for evaluating molecular mechanisms. Alves et al. identified five novel compounds (H-8, GW 5074, propentofylline, pinacidil, and SQ 22,536) with increased osteogenic activity of human MSCs using the Lopack^1280^ library based on their ALP activity and a cell proliferation assay [36]. The compounds detected in our study differed from those detected by Alves et al., except for H-8, even though the same library was used. Therefore, cell sources, detection index, time point, or compound concentration might influence the screening results. Therefore, to discover novel compounds using such screening systems, it is important to select suitable cell sources considering running costs, while conducting examinations under optimized conditions to detect the screening indices using easier methods.

The library screening using the present assay identified particularly interesting osteogenic inducible compounds, including Lef, LFM, and 1-5. Lef is a malononitrile derivative that inhibits dihydroorotate dehydrogenase and several protein tyrosine kinases [43,44]. Although Lef is an immunomodulatory agent used to treat rheumatoid arthritis [45], there have been no studies on the effects of Lef on osteoblastic differentiation. Malviya et al. reported that 15 µM Lef inhibited the proliferation of primary human osteoblasts [46]. Similarly, in the present study, 25–50 µM Lef significantly decreased the proliferation of MC3T3-E1 cells. The slight discrepancy in the inhibitory concentration of Lef on the cell proliferation likely resulted from differences in cell types, because the reactivity of primary cells/cell lines to Lef differs in the same species and among the carcinoma cell lines [46,47,48]. In this study, 1–50 µM Lef did not show cytotoxicity on MC3T3-E1 cells. Our results showed, for the first time, the positive effects of Lef on osteoblastic differentiation. Lef is associated with the Janus kinase (JAK)/signal transducer and activator of the transcription (STAT) signaling pathway [49]. The JAK/STAT pathway regulates osteogenic differentiation by activating STAT5b in osteoblasts and bone marrow-derived MSCs [50]. Although speculative, the enhanced osteogenic differentiation by Lef in the present study might be due to the activation of the JAK/STAT pathway. Further studies are required to investigate the mechanisms underlying these effects.

LFM is a potent and selective inhibitor of Bruton’s tyrosine kinase (Btk) [51,52,53]. Btk suppresses the osteogenic differentiation of MC3T3-E1 cells, primary calvarial osteoblasts, and bone marrow stromal ST2 cells [54], which supports the present results. Btk regulates osteoblastic differentiation through the MAPK, NF-κB, and protein kinase C (PKC) α signaling pathways [54]. In addition, Btk is a negative regulator of Wnt–β-catenin signaling in B cells [55]. The Wnt signaling pathway plays an important role in promoting osteogenic differentiation [56]. The osteogenic effects of LFM in the present study might involve these signaling pathways because of Btk inhibition.

1-5 is an isomer of H-7 dihydrochloride, which is a potent and selective inhibitor of PKC [57]. This family comprises more than 10 isoforms that regulate apoptosis isoforms specifically [58]. Recent studies have shown that the PKC family inhibits osteogenic differentiation [59], whereas a PKC α inhibitor promotes osteogenic differentiation via the p44/42 MAPK signaling pathway [60], which may partly explain our data.

In the present study, Lef preferentially promoted the in vitro osteogenic differentiation of rMSCs compared to LFM and 1-5; therefore, the effects of Lef on in vivo bone regeneration were investigated using a rat calvarial defect model. H&E staining showed that Lef effectively repaired calvarial bone defects, which was supported by the existence of cement lines in the area of newly formed bone, indicating active bone metabolism [28]. In addition, micro-CT analysis showed that bone mass, mineral density, and volume significantly increased following Lef administration, suggesting that Lef promotes bone regeneration. Immune response and enhanced osteoclast activity caused by inflammation affect bone resorption during bone remodeling [61]. Lef is an effective compound targeting not only osteoblasts, but also osteoclasts, and inflammatory T cells because it inhibits osteoclastogenesis [62] and activates T lymphocytes [63]. In the present study, a histological analysis using TRAP staining showed that Lef did not significantly affect the number of osteoclasts. Although Lef has the potential to regulate osteoblastic and osteoclast differentiation in vivo, the concentration of Lef used in the present study preferentially affected osteogenesis rather than osteoclastogenesis during new bone formation. It is important to analyze the effects of Lef on immune cells, such as T cells, during bone regeneration.

## 5. Conclusions

The cell-based double-screening method using the Col-1a1/GFP reporter and ALP staining assays could reliably identify candidates for osteogenesis-targeting compounds compared to conventional methods. The small-molecule compounds Lef, LFM, and 1-5 detected using this screening system promoted osteogenic differentiation, with Lef particularly promoting bone regeneration in a rat calvarial defect model, highlighting this screening method as a promising tool for identifying the novel synthetic regulators of osteogenesis.

## Figures and Tables

**Table 1:** List of candidate compounds to enhance ALP activity of MC3T3-E1 cells in the Lopack^1280^ library.

Rack Number	Rack Position	Molecular Weight	Reagent Name	Description
Strongly induced ALP activity				
9	C3	364.3	1-(5-Isoquinolinylsulfonyl)-3-methylpiperazine dihydrochloride (1-5)	Protein kinase C (PKC) inhibitor
13	B3	161.08	Putrescine dihydrochloride	Binds to the polyamine modulatory site of the NMDA glutamate receptor and potentiates NMDA-induced currents; precursor of spermidine
9	A10	270.21	Leflunomide (Lef)	Immunosuppressive; its metabolite, a malononitrile derivative, inhibits dihydroorotate dehydrogenase (in the de novo pyrimidine synthesis pathway) and several protein tyrosine kinase
9	A11	360.01	LFM-A13 (LFM)	Potent and selective inhibitor of Bruton tyrosine kinase (Btk)
11	C3	338.26	H-8 dihydrochloride (H-8)	Potent inhibitor of cAMP- and cGMP-dependent protein kinase
14	A8	254.63	Spermidine trihydrochloride	Binds to the polyamine modulatory site of the NMDA glutamate receptor
Moderately induced ALP activity				
14	A5	277.21	Ro 41-0960	Specific, reversible, orally active COMT-inhibitor
10	D7	459.3	MRS 2179	Competitive P2Y1 receptor antagonist
11	B2	195.22	L-alpha-Methyl-p-tyrosine	Tyrosine hydroxylase inhibitor
14	C8	398.73	SKF 83959 hydrobromide	Atypical D1 dopamine receptor agonist; displays antagonist activity in vitro and agonist activity in vivo
8	F8	402.45	L-165, 041	Peroxisome proliferator-activated receptor gamma agonist

## Data Availability

The datasets generated and/or analyzed during the current study are available from the corresponding authors on reasonable request.

## Supplementary Materials

The following supporting information can be downloaded at: https://www.mdpi.com/article/10.3390/biomedicines10020426/s1, Table S1: List of compounds in the Lopack^1280^ (Sigma) library, Table S2: Primers used for SYBR-based real-time quantitative RT-PCR analyses, Figure S1: Effects of identified candidate compounds on cytotoxicity and cell proliferation, Figure S2: Histological analysis for the effects of leflunomide (Lef) on osteoclast formation in the rat calvarial bone defect.
