# Supplementary material for: Cell-Based Double-Screening Method to Identify a Reliable Candidate for Osteogenesis-Targeting Compounds
Source: Biomedicines. 2022 Feb 11;10(2):426. doi: 10.3390/biomedicines10020426 (PMC8962348; doi:10.3390/biomedicines10020426)
Supplement: Supplementary file 1 [file biomedicines-10-00426-s001.zip › biomedicines-1555200-supplementary.pdf]

Cell-based double-screening method to identify a reliable candidate for osteogenesis-targeting compounds

Sho Fukuyasu, Hiroki Kayashima, Akihito Moribayashi, Shu Matsuoka, Atsuhiro Nagasaki, Hiroko Okawa, Hirofumi Yatani, Makio Saeki, Hiroshi Egusa

Supplementary Materials and Methods

Supplementary Table S1

List of compounds in the Lopack1280 (Sigma) library

Rack No	Rack Pos	Name	Sec Name	Rack No	Rack Pos	Name	Sec Name
01	A02	DL-alpha-Methyl-p-tyrosine	4-Hydroxy-alpha-methylphenylalanine	09	A02	ML-7	1-(5-Iodonaphthalene-1-sulfonyl)-1H-hexahydro-1,4-diazepine hydrochloride
01	A03	6-Methoxy-1,2,3,4-tetrahydro-9H-pyrido[3,4b] indole		09	A03	3-Isobutyl-1-methylxanthine	IBMX
01	A04	Acetamide	Amide C2	09	A04	Iproniazid phosphate	
01	A05	Amantadine hydrochloride	Tricyclo[3.3.1.1 3,7]decan-1-amine hydrochloride	09	A05	m-Iodobenzylguanidine hemisulfate	MIBG
01	A06	GABA	gamma-Aminobutyric acid	09	A06	Imetit dihydrobromide	S-[2-(Imidazol-4-yl)ethyl]isothiourea dihydrochloride
01	A07	Gabaculine hydrochloride		09	A07	JL-18	8-Methyl-6-(4-methyl-1-piperazinyl)-11H-pyrido[2,3-b][1,4]benzeodiazepine
01	A08	O-(Carboxymethyl)hydroxylamine hemihydrochloride	Aminooxyacetic acid; (Carboxymethoxy)amine hemihydrochloride	09	A08	Kenpaullone	NSC 664704
01	A09	(±)-2-Amino-7-phosphonoheptanoic acid	(±)-AP-7	09	A09	LY-367,265	1-[2-[4-(6-fluoro-1H-indol-3-yl)-3,6-dihydro-1(2H)-pyridinyl]ethyl]-5,6-dihydro-1H,4H-[1,2,5]thiadiazolo[4.3.2-ij]quinoline-2,2-dioxide
01	A10	N-Acetylprocainamide hydrochloride	Acedainide; N-Acetylnovocainamide hydrochloride; NAPA	09	A10	Leflunomide	5-Methylisoxazole-4-(4-trifluoromethylcarboxanilide)
01	A11	Actinonin	3-[[1-[(2-(Hydroxymethyl)-1-pyrrolidinyl)carbonyl]-2-methylpropyl]carbamoyl]octanohydroxamic acid	09	A11	LFM-A13	alpha-Cyano-beta-hydroxy-beta-methyl-N-(2,5-dibromophenyl)propenamide
01	B02	N-Phenylanthranilic acid	Diphenylamine-2-carboxylic acid; DPC	09	B02	(±)-Ibotenic acid	(±)-alpha-Amino-3-hydroxy-5-isoxazoleacetic acid
01	B03	S-(4-Nitrobenzyl)-6-thioguanosine	2-Amino-6-[(4-Nitrobenzyl)thio]-9-beta-D-ribofuranosylpurine	09	B03	Idazoxan hydrochloride	RX 781094
01	B04	Org 24598 lithium salt	R-(-)-N-Methyl-N-[3-[(4-trifluoromethyl)phenoxy]-3-phenyl-propyl]glycine lithium salt	09	B04	S(+)-Isoproterenol (+)-bitartrate	
01	B05	Aminophylline ethylenediamine	Theophylline ethylenediamine	09	B05	S(+)-Ibuprofen	S-(+)-2-(4-Isobutylphenyl)propionic acid
01	B06	3'-Azido-3'-deoxythymidine	Azidothymidine; AZT	09	B06	1,5-Isoquinolinediol	1,5-Dihydroxyisoquinoline

01	B07	AC 915 oxalate	N-(2-(3,4-dichlorophenyl)acetoxy)ethylpyrrolidine oxalate	09	B07	Kainic acid	2-Carboxy-3-carboxymethyl-4-isopropenylpyrrolidine
01	B08	5-(N,N-Dimethyl)amiloride hydrochloride	3-Amino-N-(aminoiminoethyl)-5-(dimethylamino)-6-chloropyrazinecarboxamide hydrochloride; DMA	09	B08	U-73343	1-[6-[((17ß)-3-Methoxyestra-1,3,5[10]-trien-17-yl)amino]hexyl]-2,5-pyrrolidinedione
01	B09	(±)-2-Amino-5-phosphonopentanoic acid	(±)-AP-5; (±)-AP-V	09	B09	LY-310,762 hydrochloride	
01	B10	Sodium Taurocholate hydrate	3alpha,7alpha,12alpha-Trihydroxy-5beta-cholan-24-oic-acid N-(2-sulfoethyl)amide	09	B10	VER-3323 hemifumarate salt	(S)-1-(6-Bromo-2,3-dihydroindol-1-yl)-2-propylamine hemifumarate salt
01	B11	Methotrexate hydrate	Methylaminopterin; MTX	09	B11	NNC 55-0396	(1S,2S)-2-(2-(N-[(3-Benzimidazol-2-yl)propyl]-N-methylamino)ethyl)-6-fluoro-1,2,3,4-tetrahydro-1-isopropyl-2-naphtyl cyclopropanecarboxylate dihydrochloride
01	C02	S(-)-p-Bromotetramisole oxalate	R 30402 oxalate	09	C02	Ifenprodil tartrate	alpha-(4-Hydroxyphenyl)-beta-(4-benzylpiperidin-1-yl)-beta-methylethanol tartrate
01	C03	TMB-8 hydrochloride	8-(Diethylamino)octyl 3,4,5-trimethoxybenzoate hydrochloride	09	C03	1-(5-Isoquinolinylsulfonyl)-3-methylpiperazine dihydrochloride	Iso-H-7
01	C04	L-azetidine-2-carboxylic acid	(S)-Azetidine-2-carboxylic acid	09	C04	L-N6-(1-Iminoethyl)lysine hydrochloride	L-NIL
01	C05	S-(p-Azidophenacyl)glutathione		09	C05	p-Iodoclonidine hydrochloride	2-[(2,6-Dichloro-4-iodophenyl)imino]imidazoline hydrochloride
01	C06	Acetyl-beta-methylcholine chloride	Methacholine chloride	09	C06	Molindone hydrochloride	3-Ethyl-1,5,6,7-tetrahydro-2-methyl-5-(4-morpholinylmethyl)-4H-indol-4-one hydrochloride
01	C07	AA-861	2-(12-Hydroxydodeca-5,10-diynyl)-3,5,6-trimethyl-p-benzoquinone	09	C07	Ketoconazole	cis-1-Acetyl-4-[4-[[2-(2,4-dichlorophenyl)-2-(1H-imidazol-1-ylmethyl)-1,3-dioxolan-4-yl]methoxy]phenyl]-piperazine
01	C08	Azathioprine		09	C08	L-701,324	7-Chloro-4-hydroxy-3-(3-phenoxy)phenylquinolin-2[1H]-one
01	C09	L-732,138	N-Acetyl-L-tryptophan 3,5-bis(trifluoromethyl)benzyl ester	09	C09	L-368,899 hydrochloride	1-((7,7-Dimethyl-2(S)-(2(S)-amino-4-(methylsulfonyl)butyramido)bicyclo[2,2,1]heptan-1(S)-yl)methylsulfonyl)-4-(2-methylphenyl)piperazine hydrochloride
01	C10	Amifostine	2-(3-Aminopropyl)aminoethyl phosphorothioate; WR2721	09	C10	Lidocaine hydrochloride	
01	C11	Atropine methyl bromide		09	C11	Ro 90-7501	2'-(4-Aminophenyl)-[2,5'-bi-1H-benzimidazol]-5-amine
01	D02	5-Aminovaleric acid hydrochloride	5-Aminopentanoic acid hydrochloride	09	D02	Isotharine mesylate	4-[1-Hydroxy-2-[(1-methylethyl)amino]butyl]-1,2-benzenediol mesylate

01	D03	4-Aminopyridine		09	D03	(-)-Isoproterenol hydrochloride	(-)-Isoprenaline hydrochloride
01	D04	p-Aminoclonidine hydrochloride	Apraclonidine hydrochloride	09	D04	3-Iodo-L-tyrosine	S(-)-3-Iodo-4-hydroxyphenylalanine
01	D05	Aminopterin	4-Aminofolic acid	09	D05	R(+)-IAA-94	R(+)-Methylindazone; Indanyloxyacetic acid 94
01	D06	5-azacytidine	4-Amino-1-(beta-D-ribofuranosyl)-1,3,5-triazin-2(1H)-one; Ladakamycin	09	D06	IB-MECA	1-Deoxy-1-[6-[[(3-iodophenyl) methyl]amino]-9H-purin-9-yl]-N-methyl-beta-D-ribofuranuronamide
01	D07	9-Amino-1,2,3,4-tetrahydroacridine hydrochloride	Tacrine hydrochloride; THA hydrochloride	09	D07	Ketorolac tris salt	Toradol
01	D08	Acyclovir	Acycloguanosine	09	D08	loxoprofen	Koloxo
01	D09	Acetylsalicylic acid	O-Acetylsalicylic acid; Aspirin	09	D09	Lomefloxacin hydrochloride	
01	D10	Acetazolamide	N-[5-(Aminosulfonyl)-1,3,4-thiadiazol-2-yl]acetamide	09	D10	Lidocaine N-ethyl bromide quaternary salt	QX-314
01	D11	Amperozide hydrochloride	4-[4,4-bis(4-Fluorophenyl)butyl]-N-ethyl-1-piperazinecarboxamide hydrochloride	09	D11	Loratadine	4-(8-chloro-5,6-dihydro-11H-benzo[5,6]cycloheptal[1,2-b]pyridin-11-ylidene-1-piperidinecarboxylic acid ethyl ester
01	E02	(±)-Nipecotic acid	(±)-3-Piperidine carboxylic acid	09	E02	Isoliquiritigenin	2',4,4'-Trihydroxychalcone
01	E03	Atropine sulfate		09	E03	1-(5-Isoquinolinylsulfonyl)-2-methylpiperazine dihydrochloride	H-7 dihydrochloride
01	E04	3-aminobenzamide	3-ABA; 3-AB	09	E04	Cibenzoline succinate	2-(2,2-diphenylcyclopropyl)-4,5-dihydro-1H-imidazole succinate
01	E05	N-Acetyl-5-hydroxytryptamine	N-Acetylserotonin; Normelatonin	09	E05	Indatraline hydrochloride	Lu 19-005
01	E06	5-(N-Ethyl-N-isopropyl)amiloride	EIPA	09	E06	Aurothioglucose	1-Thio-D-glucopyranose gold salt
01	E07	10058-F4	5-[(4-Ethylphenyl)methylene]-2-thioxo-4-thiazolidinone	09	E07	Ketoprofen	2-(3-Benzoylphenyl)propionic acid
01	E08	Amiprilose hydrochloride	1,2-O-Isopropylidene-3-O-[3'-(N,N-dimethylamino)propyl]-alpha-D-glucofuranose hydrochloride	09	E08	Labetalol hydrochloride	2-Hydroxy-5-(1-hydroxy-2-[(1-methyl-3-phenylpropyl)amino]ethyl)benzamide hydrochloride
01	E09	5-(N-Methyl-N-isobutyl)amiloride	MIA	09	E09	Lamotrigine	GI 267119X; 6-(2,3-dichlorophenyl)-1,2,4-triazine-3,5-diamine
01	E10	Arecoline hydrobromide	1-Methyl-1,2,5,6-tetrahydro-3-pyridinecarboxylic acid methyl ester hydrobromide	09	E10	L-Leucinethiol, oxidized dihydrochloride	Dithiobis(2-amino-4-methylpentane)
01	E11	Aminoguanidine hemisulfate	Hydrazinecarboximidamide hemisulfate	09	E11	(-)-Tetramisole hydrochloride	Levamisole hydrochloride
01	F02	Azelaic acid	Dicarboxylic acid C9; Nonanedioic acid; AZA	09	F02	(±)-Ibuprofen	alpha-Methyl-4-(isobutyl)phenylacetic acid
01	F03	Atropine methyl nitrate	AMN; Methylatropine nitrate	09	F03	Indomethacin	
01	F04	(±)-Norepinephrine (+)bitartrate	(±)-Arterenol (+)bitartrate; (±)-Noradrenalin (+)bitartrate	09	F04	Ivermectin	MK-933
01	F05	Aurintricarboxylic acid	ATA	09	F05	Iofetamine hydrochloride	N-Isopropyl-p-iodoamphetamine hydrochloride

01	F06	3-Aminopropionitrile fumarate		09	F06	3-(1H-Imidazol-4-yl)propyl di(p-fluorophenyl)methyl ether hydrochloride	
01	F07	1-Aminobenzotriazole	ABT; 1-Benzotriazolamine	09	F07	K 185	N-Butanoyl 2-(5,6,7-trihydro-11-methoxybenzo[c]cyclohept[2,1-a]indol-13-yl)ethanamine
01	F08	Sandoz 58-035	3-[Decyldimethylsilyl]-N-[2-(4-methylphenyl)-1-phenethyl]propanamide	09	F08	L-162,313	(5,7-dimethyl-2-ethyl-3-[[4-[2(n-butyloxycarbonylsulfonamido)-5-isobutyl-3-thienyl]phenyl]methyl]imidazo[4,5,6]pyridine
01	F09	Acetylthiocholine chloride		09	F09	alpha-Lobeline hydrochloride	(-)-Lobeline hydrochloride
01	F10	A-315456	N-[3-(cyclohexylidene(1H-imidazol-4-ylmethyl)phenyl]ethanesulfonamide	09	F10	LE 300	7-Methyl-6,7,8,9,14,15-hexahydro-5H-benz[d]indolo[2,3-g]azecine
01	F11	Agmatine sulfate	(4-Aminobutyl)guanadine sulfate	09	F11	L-655,708	Ethyl (S)-11,12,13,13a-Tetrahydro-7-methoxy-9-oxo-9H-imidazo[1,5-a]pyrrolo[2,1-c][1,4]benzodiazepine-1-carboxylate
01	G02	Tryptamine hydrochloride	3-(2-Aminoethyl)indole hydrochloride	09	G02	SD-169	Indole-5-carboxamide
01	G03	Arcaine sulfate	N,N'-1,4-Butanediylbis(guanidine) sulfate	09	G03	Imipramine hydrochloride	
01	G04	4-Amino-1,8-naphthalimide		09	G04	Imiloxan hydrochloride	RS 21361
01	G05	(±)-2-Amino-4-phosphonobutyric acid	(±)-AP-4	09	G05	ICI 204,448 hydrochloride	
01	G06	Apigenin	4',5,7-Trihydroxyflavone	09	G06	Isonipecotic acid	4-Piperidine carboxylic acid
01	G07	3-Amino-1-propanesulfonic acid sodium		09	G07	Ketotifen fumarate	
01	G08	(±)-2-Amino-3-phosphonopropionic acid	(±)-AP-3	09	G08	CyPPA	Cyclohexyl-[2-(3,5-dimethyl-pyrazol-1-yl)-6-methyl-pyrimidin-4-yl]-amine
01	G09	4-Androsten-4-ol-3,17-dione	4-OH-A; 4-Hydroxy-4-androstene-3,17-dione	09	G09	Loperamide hydrochloride	
01	G10	GR 46611	3-[3-(2-Dimethylaminoethyl)-1H-indol-5-yl]-N-(4-methoxybenzyl)acrylamide	09	G10	Lansoprazole	
01	G11	4-Aminobenzamidine dihydrochloride		09	G11	LY-294,002 hydrochloride	2-(4-Morpholinyl)-8-phenyl-4H-1-benzopyran-4-one hydrochloride
01	H02	5-Fluoroindole-2-carboxylic acid		09	H02	(±)-Isoproterenol hydrochloride	
01	H03	1-Aminocyclopropanecarboxylic acid hydrochloride	ACPC	09	H03	Isoxanthopterin	2-Amino-4,7-dihydroxypteridine
01	H04	Reserpine	Methyl reserpate; 3,4,5-Trimethoxybenzoic acid ester	09	H04	Stevioside	(4a-13-[(2-O-ß-D-Glucopyranosyl-ß-D-glucopyranosyl)oxy]kaur-16-en-18-oic acid ß-D-glucopyranosyl ester
01	H05	N-arachidonylglycine	NAGly	09	H05	ICI 118,551 hydrochloride	(±)-1-[2,3-(Dihydro-7-methyl-1H-inden-4-yl)oxy]-

							3-[(1-methylethyl)amino]-2-butanol hydrochloride
01	H06	(+)-Butaclamol hydrochloride		09	H06	JWH-015	(2-Methyl-1-propyl-1H-indol-3-yl)-1-naphthalenylmethanone
01	H07	Apomorphine hydrochloride hemihydrate	10,11-Dihydroxyaporphine hydrochloride hemihydrate	09	H07	Kynurenic acid	4-Hydroxyquinoline-2-carboxylic acid
01	H08	L-Arginine		09	H08	beta-Lapachone	
01	H09	2-(2-Aminoethyl)isothiourea dihydrobromide	AET; S-(2-Aminoethyl)isothiouronium dihydrobromide	09	H09	Lonidamine	Diclondazolic acid
01	H10	2-Hydroxysaclofen	(±)-3-Amino-2-(4-chlorophenyl)-2-hydroxy-propylsulfonic acid	09	H10	L-687,384 hydrochloride	1'-Benzyl-3,4-dihydrospiro[naphthalene-1-(2H),4'-piperidine] hydrochloride
01	H11	3-Aminopropylphosphonic acid		09	H11	Loxapine succinate	
02	A02	N-Acetyl-L-Cysteine		10	A02	TMPH hydrochloride	2,2,6,6-Tetramethylpiperidin-4-yl heptanoate hydrochloride
02	A03	6-Aminohexanoic acid	6-Aminocaproic acid; EACA	10	A03	L-750,667 trihydrochloride	(±)-3-[4-Iodophenyl)-1-piperazyl] methylpyrrolo [2,3-b] pyrimidine
02	A04	Altretamine	N,N,N',N',N'',N''-Hexamethyl-1,3,5-triazine-2,4,6-triamine	10	A04	4-Methylpyrazole hydrochloride	Fomepizole
02	A05	Adenosine 3',5'-cyclic monophosphate	cAMP; 3',5'-Cyclic AMP	10	A05	p-MPPI hydrochloride	4-Iodo-N-[2-4-(methoxyphenyl)-1-piperazinyl]ethyl]-N-2-pyridinyl-benzamide hydrochloride
02	A06	(±)-AMT hydrochloride	(±)-2-Amino-5,6-dihydro-6-methyl-4H-1,3-thiazine hydrochloride	10	A06	Molsidomine	SIN-10
02	A07	5'-N-Methyl carboxamidoadenosine	MECA	10	A07	Metergoline	[[(8beta)-1,6-Dimethylergolin-8-yl]-methyl]carbamic acid phenylmethyl ester
02	A08	1-Allyl-3,7-dimethyl-8-p-sulfophenylxanthine		10	A08	Meclofenamic acid sodium	2-([2,6-Dichloro-3-methylphenyl]amino)benzoic acid sodium
02	A09	Acetohexamide		10	A09	(±)-Metoprolol (+)-tartrate	1-(Isopropylamino)-3-(p-[beta-methoxyethyl]phenoxy)-2-propanol tartrate
02	A10	cis-Azetidine-2,4-dicarboxylic acid		10	A10	GW405833 hydrochloride	1-(2,3-Dichlorobenzoyl)-5-methoxy-2-methyl-(3-(morpholin-4-yl)ethyl)-1H-indole hydrochloride
02	A11	2,3-Butanedione monoxime	Diacetyl monoxime	10	A11	MDL 28170	Z-Val-Phe-CHO
02	B02	L-2-aminoadipic acid	Aad; (S)-2-Aminohexanedioic acid; L-Homoglutamic acid	10	B02	Lorglumide sodium	CR 1409
02	B03	ATPO	(R,S)-2-Amino-3-[5-tert-butyl-3-(phosphonomethoxy)-4-isoxazolyl]propionic acid	10	B03	Linopirdine	DuP 996
02	B04	2-(Methylthio)adenosine 5'-diphosphate trisodium salt hydrate	2-Methylthio-ADP trisodium salt hydrate	10	B04	Nocodazole	R 17934
02	B05	L(-)-Norepinephrine bitartrate	(-)-Arterenol bitartrate; Noradrenaline bitartrate	10	B05	Metaproterenol hemisulfate	

02	B06	Paroxetine hydrochloride hemihydrate (MW = 374.83)	(3S-trans)-3-[(1,3-benzodioxol-5-yloxy)methyl]-4-(4-fluorophenyl)piperadine hydrochloride hemihydrate	10	B06	3-Methyl-6-(3-[trifluoromethyl]phenyl)-1,2,4-triazolo[4,3-b]pyridazine	CL 218,872
02	B07	PNU-37883A	N-(1-Adamantyl)-N'-cyclohexyl-4-morpholinecarboxamidine hydrochloride	10	B07	(-)-cis-(1S,2R)-U-50488 tartrate	(-)-(1S,2R)-cis-3,4-Dichloro-N-methyl-N-[2-(1-pyrrolidinyl)cyclohexyl]benzeneacetamide tartrate
02	B08	trans-(±)-ACPD	trans-(±)-1-Amino-1,3-cyclopentanedicarboxylic acid	10	B08	Milrinone	1,6-Dihydro-2-methyl-6-oxo-[3,4'-bipyridine]-5-carbonitrile
02	B09	SKF-89145 hydrobromide	4-(3,4-Dihydroxyphenyl)-6-methyl-4,5,6,7-tetrahydro-thieno[2,3-c]pyridine hydrobromide	10	B09	6-Methyl-2-(phenylethynyl)pyridine hydrochloride	MPEP hydrochloride
02	B10	trans-Azetidine-2,4-dicarboxylic acid	tADA	10	B10	2-methoxyestradiol	2-Hydroxyestradiol 2-methyl ether
02	B11	SB 222200		10	B11	Myricetin	Cannabiscetin
02	C02	N-Acetyltryptamine	3-(2-N-Acetylaminoethyl)indole	10	C02	PM-20	1-[1,1'-biphenyl]-4-yl-3,4-bis[(2-hydroxyethyl)thio]-1H-Pyrrole-2,5-dione
02	C03	Allopurinol	1H-Pyrazolo[3,4-d]pyrimidin-4-ol	10	C03	L-741,626	(±)-3-[4-(4-Chlorophenyl)-4-hydroxypiperidinyl]methylindole
02	C04	Aminoguanidine hydrochloride	Guanylhydrazine hydrochloride	10	C04	N-omega-Methyl-5-hydroxytryptamine oxalate salt	N-omega-Methylserotonin
02	C05	5-(N,N-hexamethylene)amiloride		10	C05	Mianserin hydrochloride	1,2,3,4,10,14b-Hexahydro-2-methyldibenzo[c,f]pyryzino[1,2-a]azepine hydrochloride
02	C06	Antozoline hydrochloride	2-(N-Benzylanilinomethyl)-2-imidazoline hydrochloride	10	C06	Mizoribine	N'-(beta-D-Ribofuranosyl)-5-hydroxyimidazole-4-carboxamide
02	C07	Tracazolate	4-(Butylamino)-1-ethyl-6-methyl-1H-pyrazolo [3,4-b]pyridine-5-carboxylic acid ethyl ester	10	C07	Clorgyline hydrochloride	N-Methyl-N-propargyl-3-(2,4-dichlorophenoxy)-propylamine hydrochloride
02	C08	Diacylglycerol kinase inhibitor I	R 59022	10	C08	(±)-alpha-Methyl-4-carboxyphenylglycine	(±)-MCPG
02	C09	cis-4-Aminocrotonic acid	CACA	10	C09	Mibefradil dihydrochloride	Ro 40-5967; (1S,2S)-2-[2[[3-(2-benzimidazolylpropyl]methylamino]ethyl]-6-fluoro-1,2,3,4-tetrahydro-1-isopropyl-2-naphthyl methoxyacetate dihydrochloride
02	C10	CBIQ	4-Chlorobenzo[f]isoquinolone	10	C10	Cysteamine hydrochloride	Mercaptamine; MEA hydrochloride
02	C11	1-benzoyl-5-methoxy-2-methylindole-3-acetic acid		10	C11	NG-Monomethyl-L-arginine acetate	L-NMMA
02	D02	Amiloride hydrochloride		10	D02	cis(+/-)-8-OH-PBZI hydrobromide	cis-8-Hydroxy-3-(n-propyl)-1,2,3a,4,5,9b-hexahydro-1H-benz[e]indole hydrobromide
02	D03	Amitriptyline hydrochloride		10	D03	L-733,060 hydrochloride	(2S,3S) 3-([3,5-Bis(trifluoromethyl)phenyl]methoxy)-2-phenylpiperidine hydrochloride
02	D04	BW 284c51	1,5-Bis(4-allyldimethylammoniumphenyl)pentan-3-one dibromide	10	D04	Moxonidine hydrochloride	BDF-5895
02	D05	Fulvestrant	(7a, 17b)-7-[9-[(4,4,5,5,5-pentafluoropentyl)Sulfinyl]n	10	D05	Mevastatin	Compactin

			onyl]estra-1,3,5-(10)-triene-3,17-diol				
02	D06	Aniracetam		10	D06	S-Methylisothiourea hemisulfate	Carbamimidothioic acid methyl ester hemisulfate
02	D07	Amoxapine		10	D07	MRS 2179	2'-Deoxy-N6-methyl adenosine 3',5'-diphosphate diammonium salt
02	D08	1-Amino-1-cyclohexanecarboxylic acid hydrochloride		10	D08	1-Methylhistamine dihydrochloride	
02	D09	N6-2-(4-Aminophenyl)ethyladenosine	APNEA	10	D09	N6-Methyladenosine	6-Methylaminopurine-9-ribofuranoside
02	D10	AIDA	1-Aminoindan-1,5-dicarboxylic acid; UPF 523	10	D10	alpha,beta-Methylene adenosine 5'-triphosphate dilithium	alpha,beta-Methylene ATP, AMP-CPP dilithium
02	D11	p-Benzoquinone	Quinone; p-BQ	10	D11	MK-912	L-657,743 hydrochloride
02	E02	(±)-Atenolol		10	E02	L-703,606 oxalate	cis-2-(Diphenylmethyl)-N-[(2-iodophenyl)methyl]-1-azabicyclo[2.2.2]octan-3-amine oxalate
02	E03	Amiodarone hydrochloride		10	E03	Metoclopramide hydrochloride	
02	E04	Adenosine		10	E04	MRS 1845	N-Propargylnitrendipene
02	E05	(±)-p-Aminoglutethimide		10	E05	8-Methoxymethyl-3-isobutyl-1-methylxanthine	8-Methoxymethyl-IBMX
02	E06	MRS 2211 sodium salt hydrate	2-[(2-Chloro-5-nitrophenyl)azo]-5-hydroxy-6-methyl-3-[(phosphonooxy)methyl]-4-pyridinecarboxaldehyde sodium salt hydrate sodium salt hydrate	10	E06	MG 624	N,N,N-Triethyl-2-(4-trans-stilbenoxy)ethylammonium iodide
02	E07	Aminobenztropine	ABT	10	E07	Meloxicam sodium	4-Hydroxy-2-methyl-N-(5-methyl-2-thiazolyl)-2H-1,2-benzothiazine-3-carboxamide 1,1-dioxide
02	E08	Alaproclate hydrochloride	D,L-Alanine, 2-(4-chlorophenyl)-1,1-dimethylethyl ester hydrochloride	10	E08	Moxisylyte hydrochloride	4-Dimethylaminoethoxy-5-isopropyl-2-methylphenyl acetate hydrochloride
02	E09	Opipramol dihydrochloride	4-[3-(5H-Dibenz[b,f]azepin-5-yl)propyl]-1-piperazineethanol dihydrochloride	10	E09	(S)-MAP4 hydrochloride	(S)-2-Amino-2-methyl-4-phosphonobutanoate hydrochloride
02	E10	A-77636 hydrochloride		10	E10	Methoxamine hydrochloride	
02	E11	8-Bromo-cGMP sodium	8-Bromoguanosine-3',5'-cyclophosphate sodium	10	E11	(±)-3-(3,4-dihydroxyphenyl)-2-methyl-DL-alanine	DL-alpha-Methyl-DOPA
02	F02	Xli 093 hydrate	Bis[8-ethynyl-5,6-dihydro-5-methyl-6-oxo-4H-imidazo[1,5-a][1,4]benzodiazepine-3-carboxylic acid] 1,3-propanediyl ester hydrate	10	F02	Levallorphan tartrate	17-(2-Propenyl)morphinan-3-ol tartrate
02	F03	4-(2-Aminoethyl)benzenesulfonyl fluoride hydrochloride	AEBSF	10	F03	R(-)-Me5	1-(2,6-Dimethylphenoxy)-3-methyl-2-butanamine hydroiodide
02	F04	L-Aspartic acid		10	F04	BIO	(2'Z,3'E)-6-Bromoindirubin-3'-oxime

02	F05	(±)-HA-966	(±)-3-Amino-1-hydroxy-2-pyrrolidone	10	F05	MK-886	3-[3-tert-Butylthio-1-(4-chlorobenzyl)-5-isopropyl-1H-indol-2-yl]-2,2-dimethylpropionic acid, sodium salt
02	F06	8-(p-Sulfophenyl)theophylline		10	F06	N-Methyl-D-aspartic acid	NMDA
02	F07	Arecaidine propargyl ester hydrobromide	APE	10	F07	Morin	2',3,4',5,7-Pentahydroxyflavone
02	F08	Psora-4	5-(4-Phenylbutoxy)psoralen	10	F08	S-Methyl-L-thiocitrulline acetate	N5-[Imino(methylthio)methyl]-L-ornithine acetate
02	F09	gamma-Acetylinic GABA	4-Amino-5-hexynoic acid	10	F09	(±)-Methoxyverapamil hydrochloride	D600; Gallopamil
02	F10	ATPA	(RS)-2-Amino-3-(3-hydroxy-5-tert-butylisoxazol-4-yl)propanoic acid	10	F10	Mitoxantrone	1,4-Dihydroxy-5,8-bis([2-([2-hydroxyethyl]amino)ethyl]amino)-9,10-anthracenedione
02	F11	TBBz	4,5,6,7-Tetrabromobenzimidazole	10	F11	MRS 2159	
02	G02	L-allylglycine	L-2-Amino-4-pentenoic acid	10	G02	AFMK	N-[3-[2-(Formylamino)-5-methoxyphenyl]-3-oxopropyl]acetamide; NSC 688263
02	G03	Ancitabine hydrochloride	Cyclocytidine hydrochloride; Cyclo-C	10	G03	Dihydrocapsaicin	8-Methyl-N-vanillylnonanamide
02	G04	Astaxanthin	3,3'-Dihydroxy-beta,beta-carotene-4,4'-dione	10	G04	MRS 1523	3-propyl-6-ethyl-5-[(ethylthio)carbonyl]-2-phenyl-4-propyl-3-pyridine carboxylate
02	G05	Lercanidipine hydrochloride hemihydrate		10	G05	Mexiletene hydrochloride	1-(2,6-Xylyloxy)-2-aminopropane
02	G06	1,3-Dipropyl-8-p-sulfophenylxanthine		10	G06	alpha-Methyl-DL-tyrosine methyl ester hydrochloride	AMPT
02	G07	Indirubin-3'-oxime		10	G07	Minoxidil	
02	G08	SB 200646 hydrochloride	N-(1-Methyl-1H-indol-5-yl)-N'-3-pyridinyl-urea hydrochloride	10	G08	Melatonin	N-[2-(5-Methoxyindol-3-yl)ethyl]acetamide
02	G09	AB-MECA	N6-(4-Aminobenzyl)-9-[5-(methylcarbonyl)-beta-D-ribofuranosyl]adenine	10	G09	Metrazoline oxalate	1H-Imidazole, 4,5-dihydro-2-[(1E)-2-(2-methylphenyl)ethenyl,-ethandioate
02	G10	ARL 67156 trisodium salt	FPL 67156	10	G10	O-Methylserotonin hydrochloride	Mexamine hydrochloride
02	G11	Bromoenol lactone	BEL; E-6-(Bromoethylene)tetrahydro-3-(1-naphthyl)-2H-pyran-2-one	10	G11	GR 127935 hydrochloride hydrate	N-[4-Methoxy-3-(4-methyl-1-piperazinyl)phenyl]-2'-methyl-4'-(5-methyl-1,2,4-oxadiazol-3-yl)-1,1'-biphenyl-4-carboxamide hydrochloride
02	H02	ABT-418 hydrochloride	3-Methyl-5-[(2S)-1-methyl-2-pyrrolidinyl]isoxazole hydrochloride	10	H02	L-745,870 hydrochloride	3-[[4-(4-Chlorophenyl)piperazin-1-yl]methyl]-1H-pyrrolo[2,3-b]pyridine hydrochloride
02	H03	Alprenolol hydrochloride		10	H03	(-)-Naproxen sodium	(S)-6-Methoxy-alpha-methyl-2-naphthaleneacetic acid sodium
02	H04	N-(4-Amino-2-chlorophenyl)phthalimide		10	H04	Melphalan	L-Phenylalanine mustard
02	H05	Amsacrine hydrochloride	m-AMSA hydrochloride	10	H05	Methylergonovine maleate	Methergine maleate
02	H06	2-Methylthioadenosine triphosphate tetrasodium	2-Methylthio ATP tetrasodium	10	H06	ML 10302	2-Piperidinoethyl-4-amino-5-chloro-2-methoxybenzoate

02	H07	S(-)-Atenolol		10	H07	Rufinamide	1-[(2,6-difluorophenyl)methyl]-1H-1,2,3-Triazole-4-carboxamide
02	H08	D(-)-2-Amino-7-phosphonoheptanoic acid	D-AP-7	10	H08	L-Methionine sulfoximine	L-S-(3-Amino-3-carboxypropyl)-S-methylsulfoximine
02	H09	Alloxazine	Isoalloxazine	10	H09	GW9662	2-Chloro-5-nitro-N-phenyl-benzamide
02	H10	Beclomethasone	9alpha-Chloro-16beta-methyl-1,4-pregnadiene-11beta,17alpha,21-triol-3,20-dione	10	H10	Se-(methyl)selenocysteine hydrochloride	Se-MSC
02	H11	Benzamide	Benzoylamide	10	H11	2,6-Difluoro-4-[2-(phenylsulfonylamino)ethylthio]phenoxyacetamide	PEPA
03	A02	3-Bromo-7-nitroindazole		11	A02	Mifepristone	RU-486
03	A03	Bumetanide	3-(Aminosulfonyl)-5-(butylamino)-4-phenoxybenzoic acid	11	A03	Minocycline hydrochloride	
03	A04	(±)-Baclofen	Lioresal	11	A04	(-)-MK-801 hydrogen maleate	(5S,10R)-(–)-5-Methyl-10,11-dihydro-5H-dibenzo[a,d]cyclohepten-5,10-imine
03	A05	Brefeldin A from Penicillium brefeldianum	BFA; Ascotoxin, Cyanein	11	A05	Methiothepin mesylate	1-[10,11-Dihydro--8-(methylthio)dibenzo[b,f]thiepin-10-yl]-4-methylpiperazine mesylate
03	A06	BP 897	N-[4-(4-(2-methoxyphenyl)piperazinyl)butyl]-2-naphthamide	11	A06	MDL 105,519	(Z)-2-Carboxy-4,6-dichloroindole-3-(2'-phenyl-2'-carboxy)-ene
03	A07	Bupropion hydrochloride	(±)-1-(3-Chlorophenyl)-2-[(1,1-dimethylethyl)amino]-1-propanone hydrochloride	11	A07	nor-Binaltorphimine dihydrochloride	nor-BNI dihydrochloride
03	A08	BU224 hydrochloride	2-(4,5-Dihydroimidazol-2-yl)-quinoline hydrochloride	11	A08	NCS-356	4-(4-chlorophenyl)-4-hydroxy-2-butanoic acid
03	A09	Ciprofibrate	2-[p-(2,2-Dichlorocyclopropyl)phenoxy]-2-methylpropanoic acid	11	A09	(-)-Nicotine hydrogen tartrate salt	(-)-1-Methyl-2-(3-pyridyl)pyrrolidine
03	A10	CGP-7930	3-(3',5'-Di-tert-butyl-4'-hydroxy)phenyl-2,2-dimethylpropanol	11	A10	Nicardipine hydrochloride	YC-93 hydrochloride
03	A11	Chlorprothixene hydrochloride	2-Chloro-9-(3-dimethylaminopropylidene)thioxanthene hydrochloride	11	A11	NF 023	8,8'-[carbonylbis(imino-3,1-phenylene carbonylimino)]bis(1,3,5-naphthalene-trisulfonic acid) hexasodium salt
03	B02	(+)-Bromocriptine methanesulfonate		11	B02	L-alpha-Methyl-p-tyrosine	(S)-alpha-Methyltyrosine
03	B03	Betaine hydrochloride		11	B03	Maprotiline hydrochloride	9-(gamma-Methylaminopropyl)-9,10-dihydro-9,10-ethanoanthracene hydrochloride
03	B04	SB 202190	4-[4-(4-Fluorphenyl)-5-(4-pyridinyl)-1H-imidazol-2-yl]phenol	11	B04	2-Methyl-5-hydroxytryptamine maleate	2-Methylserotonin maleate
03	B05	Budesonide	16,17-Butylidenebis(oxy)-11,21-dihydroxypregna-1,4-diene-3,20-dione	11	B05	PRL-3 Inhibitor I	5-[[5-bromo-2-[(2-bromophenyl)methoxy]phenyl]methylene]-2-thioxo-4-thiazolidinone
03	B06	(E)-5-(2-Bromovinyl)-2'-deoxyuridine	BVdU	11	B06	Metrifudil	N-[(2-Methylphenyl)methyl]-adenosine

03	B07	(-)-Bicuculline methbromide, 1(S), 9(R)		11	B07	Neostigmine bromide	
03	B08	B-HT 933 dihydrochloride	Azepexole dihydrochloride	11	B08	S-Nitrosoglutathione	GSNO
03	B09	6-Chloromelatonin	N-Acetyl-6-chloro-5-methoxytryptamine	11	B09	NG-Nitro-L-arginine	L-NOARG; L-NNA
03	B10	CGP-13501	3-(3',5'-Di-tert-butyl-4'-hydroxy)phenyl-2,2-dimethylpropanal	11	B10	Nifedipine	
03	B11	Choline bromide	Choline-methyl-13C bromide	11	B11	Nimustine hydrochloride	ACNU
03	C02	O6-benzylguanine		11	C02	SB-215505	6-Chloro-5-methyl-1-5-quinolycarbamoyl-indoline
03	C03	Betaine aldehyde chloride	(Formylmethyl)trimethylammonium chloride	11	C03	H-8 dihydrochloride	N-[2-(Methylamino)ethyl]-5-isoquinolinesulfonamide dihydrochloride
03	C04	Bay 11-7085	(E)-3-(4-t-Butylphenylsulfonyl)-2-propenenitrile	11	C04	alpha-Methyl-5-hydroxytryptamine maleate	alpha-Methylserotonin maleate
03	C05	8-Bromo-cAMP sodium	8-Bromoadenosine-3',5'-cyclophosphate sodium	11	C05	Mesulergine hydrochloride	CU 32-085 hydrochloride
03	C06	BRL 15572	4-(3-Chlorophenyl)-alpha-(diphenylmethyl)-1-piperazineethanol hydrochloride	11	C06	p-MPPF dihydrochloride	4-Fluoro-N-(2-[4-(2-methoxyphenyl)1-piperazinyl]ethyl)-N-(2-pyridinyl)benzamide dihydrochloride
03	C07	(±)-Bay K 8644	1,4-Dihydro-2,6-dimethyl-5-nitro-4-[2-(trifluoromethyl)-phenyl]-<br>3-pyridine carboxylic acid methyl ester	11	C07	CR 2249	Nebostinel
03	C08	(±)-Butaclamol hydrochloride	AY 23028	11	C08	NCS-382	1-Ethyl-1,4-dihydro-7-methyl-4-oxo-1,8-naphthyridine-3-carboxylic acid
03	C09	Carmustine	BCNU; 1,3-Bis(2-chloroethyl)-1-nitrosourea	11	C09	Naphazoline hydrochloride	2-(1-Naphthylmethyl)imidazoline nitrate
03	C10	CP55940	5-(1,1-dimethylheptyl)-2-[5-hydroxy-2-(3-hydroxypropyl)cyclohexyl]phenol	11	C10	Naloxone hydrochloride	
03	C11	BTO-1	5-Cyano-7-nitro-2-benzothiazolecarboxamide-3-oxide	11	C11	Norcantharidin	
03	D02	N-Bromoacetamide	NBA	11	D02	1-Methylimidazole	Methimazole
03	D03	Benazoline oxalate	4,5-Dihydro-2-(2-naphthalenyl)-1H-imidazole oxalate	11	D03	Proglumide	4-Benzoylamino-5-dipropylamino-5-oxopentanoic acid
03	D04	Betaxolol hydrochloride		11	D04	Metolazone	
03	D05	Benztropine mesylate		11	D05	MDL 26,630 trihydrochloride	1,5-(Diethylamino)piperidine trihydrochloride
03	D06	Chloroethylclonidine dihydrochloride	CEC dihydrochloride	11	D06	Levetiracetam	(alphaS)-alpha-Ethyl-2-oxo-1-pyrrolidineacetamide
03	D07	Bromoacetylcholine bromide	2-(2-Bromoacetyloxy)-N,N,N-trimethylethanaminium bromide	11	D07	S-(4-Nitrobenzyl)-6-thioinosine	NBTI
03	D08	BRL 37344 sodium	(±)-(R*,R*)-[4-[2-[[2-(3-Chlorophenyl)-2-hydroxyethyl] amino]propyl]phenoxy]-acetic acid sodium	11	D08	Nalidixic acid sodium	1-Ethyl-1,4-dihydro-7-methyl-4-oxo-1,8-naphthyridine-3-carboxylic acid sodium
03	D09	PK 11195	1-(2-Chlorophenyl)-N-methyl-N-(1-methylpropyl)	11	D09	3-Nitropropionic acid	

03	D10	L-Cycloserine	(S)-4-Amino-3-isoxazolidone	11	D10	7-Nitroindazole	
03	D11	CB 1954	5-(1-Aziridinyl)-2,4-dinitrobenzamide	11	D11	Noscapine hydrchloride	Narcotine
03	E02	(±)-Brompheniramine maleate		11	E02	Mecamylamine hydrochloride	
03	E03	BWB70C	N-[3-[3-4(-Fluorophenoxy)phenyl]-1-methyl-2-propenyl]-N-hydroxyurea	11	E03	Fenobam	N-(3-Chlorophenyl)-N'-(4,5-dihydro-1-methyl-4-oxo-1H-imidazole-2-yl)urea
03	E04	Benzamidine hydrochloride	Amidinobenzene hydrochloride	11	E04	DFB	3,3'-Difluorobenzaldazine
03	E05	Ro 20-1724	4-[(3-Butoxy-4-methoxyphenyl)methyl]-2-imidazolidione	11	E05	ZM 39923 hydrochloride	3-(N-Benzyl-N-isopropyl)amino-1-(naphthalen-2-yl)propan-1-one hydrochloride
03	E06	6-Fluoronorepinephrine hydrochloride	6-FNE hydrochloride	11	E06	Niflumic acid	2-(3-[Trifluoromethyl]anilino)nicotinic acid
03	E07	BMY 7378 dihydrochloride	8-[2-[4-(2-Methoxyphenyl)-1-piperazinyl]ethyl-azaspiro[4.5]decane-7,9-dione dihydrochloride	11	E07	Naltrexone hydrochloride	17-(Cyclopropylmethyl)-4,5-epoxy-3,14-dihydroxymorphinan-6-one hydrochloride
03	E08	BRL 54443 maleate	3-(1-Methylpiperidin-4-yl)-1H-indol-5-ol maleate	11	E08	Gossypol	2,2'-bis(8-Formyl-1,6,7-trihydroxy-5-isopropyl-3-methylnaphthalene)
03	E09	Caffeic Acid	3,4-Dihydroxycinnamic acid	11	E09	NG-Nitro-L-arginine methyl ester hydrochloride	L-NAME hydrochloride
03	E10	ML-9		11	E10	NS 521 oxalate	1-(1-Butyl)-4-(2-oxo-1-benzimidazolinyl)piperidine oxalate
03	E11	S-(+)-PD 123177 trifluoroacetate salt hydrate	(S)-1-[(4-Amino-3-methylphenyl)methyl]-5-(diphenylacetyl)-4,5,6,7-tetrahydro-1H-Imidazo[4,5-c]pyridine-6-carboxylic acid trifluoroacetate salt hydrate	11	E11	(+)-Nicotine (+)-di-p-toluoyl tartrate	R(+)-3-(1-Methyl2-pyrrolidinyl)pyridinium (+)di-p-toluoyl tartrate
03	F02	Benzamil hydrochloride	N-(Benzylamidino)-3,5-diamino-6-chloropyrazinecarboxamide hydrochloride	11	F02	Methapyrilene hydrochloride	N,N-Dimethyl-N'-(2-pyridinyl)-N'-(2-thienylmethyl)-1,2-ethanediamine hydrochloride
03	F03	5-Bromo-2'-deoxyuridine	Br-dU; 5-Bromo-1-(2-deoxy-beta-D-ribofuranosyl)uracil	11	F03	(±)-Muscarine chloride	Tetrahydro-4-hydroxy-N,N,N,5-tetramethyl-2-furanmethanammonium chloride
03	F04	Betamethasone	9alpha-Fluoro-16beta-methylprednisolone	11	F04	L-alpha-Methyl DOPA	MK-351; Methyldopa
03	F05	Bestatin hydrochloride	N-[(2S,3R)-3-Amino-2-hydroxy-4-phenylbutyryl]-L-leucine hydrochloride	11	F05	3-Morpholinosydnonimine hydrochloride	Linsidomine hydrochloride
03	F06	Bromoacetyl alprenolol menthane		11	F06	Nimesulide	N-(4-Nitro-2-phenoxyphenyl)methanesulfonamide
03	F07	R(+)-6-Bromo-APB hydrobromide	R(+)-6-Bromo-7,8-dihydroxy-3-allyl-1-phenyl-2,3,4,5-tetrahydro-1H-3-benzazepine hydrobromide	11	F07	S-Nitroso-N-acetylpenicillamine	SNAP
03	F08	Biperiden hydrochloride	1-(5-Bicyclo[2.2.1]hept-2-enyl)-1-phenyl-3-(1-piperidinyl)propan-1-ol hydrochloride	11	F08	5-Nitro-2-(3-phenylpropylamino)benzoic acid	NPPB

03	F09	Cilostazol	OPC 13013; OPC 21; Pletaal	11	F09	(±)-Normetanephrine hydrochloride	alpha-(Aminomethyl)-4-hydroxy-3-methoxy-benzyl alcohol hydrochloride
03	F10	(+)-Catechin Hydrate	(+)-Cyanidol-3	11	F10	CI-976	2,2-Dimethyl-N-(2,4,6-trimethoxyphenyl)dodecanamide
03	F11	Corticosterone	Kendall's Compound B; 4-Pregnene-11beta,21-diol-3,20-dione; Reichstein's Substance H	11	F11	Naltrindole hydrochloride	NTI hydrochloride
03	G02	L-Buthionine-sulfoximine		11	G02	Memantine hydrochloride	3,5-Dimethyl-1-adamantanamine hydrochloride
03	G03	Bepridil hydrochloride		11	G03	Methoctramine tetrahydrochloride	N,N'-bis[6-[[(2-Methoxyphenyl)methyl]amino]hexyl]1,8-octane diamine tetrahydrochloride
03	G04	Buspirone hydrochloride		11	G04	Methysergide maleate	
03	G05	Bretylium tosylate	2-Bromo-N-ethyl-N,N-dimethylbenzenemethanaminium 4-methyl-benzenesulfontate	11	G05	3-Methoxy-morphanin hydrochloride	nor-Dextromethorphan hydrochloride
03	G06	Benoxathian hydrochloride	2-[[[2-(2,6-Dimethoxyphenoxy)ethyl]-amino]-methyl]-1,4-benzoxanthian hydrochloride	11	G06	Nialamide	4-Pyridinecarboxylic acid 2-[3-oxo-3-[(phenylmethyl)amino]propyl] hydrazide
03	G07	BTCP hydrochloride		11	G07	Niclosamide	2',5'-Dichloro-4'-nitrosalicylanilide
03	G08	Chlorambucil	4-[Bis(2-chloroethyl)amino]benzenebutyric acid	11	G08	AMN082	N,N'-Dibenzhydrylethane-1,2-diamine dihydrochloride
03	G09	Caffeine	1,3,7-Trimethylxanthine	11	G09	Nortriptyline hydrochloride	
03	G10	Chlorpropamide		11	G10	6-Nitroso-1,2-benzopyrone	
03	G11	Carboplatin	cis-Diammine(1,1-cyclobutanedicarboxylato) platinum	11	G11	Sertraline hydrochloride	(1S,4S)-4-(3,4-Dichlorophenyl)-1,2,3,4-tetrahydro-N-methyl-1-napthalenamine hydrochloride
03	H02	DL-Buthionine-[S,R]-sulfoximine		11	H02	Me-3,4-dephostatin	3,4-Dihydroxy-N-methyl-N-nitrosaline
03	H03	(+)-Brompheniramine maleate	Dexbrompheniramine maleate	11	H03	(+)-MK-801 hydrogen maleate	Dizocilipine maleate
03	H04	Benserazide hydrochloride	DL-Serine, 2-[(2,3,4-trihydroxyphenyl)methyl]hydrazide hydrochloride	11	H04	Ethopropazine hydrochloride	10-[2-(Diethylamino)propyl]phenothiazine hydrochloride
03	H05	BRL 50481	5-Nitro-2,N,N-trimethylbenzenesulfonamide	11	H05	S15535	1-(2,3-Dihydro-1,4-benzodioxin-5-yl)-4-(2,3-dihydro-1h-inden-2-yl)-piperazine
03	H06	Phenoxybenzamine hydrochloride		11	H06	Nomifensine maleate	1,2,3,4-Tetrahydro-2-methyl-4-phenyl-8-isoquinolinamine maleate
03	H07	DAPH	4,5-Dianilinophthalimide; CGP 52411	11	H07	NAN-190 hydrobromide	1-(2-Methoxyphenyl)-4-[4-(2-phthalimido)butyl]piperazine hydrobromide
03	H08	Supercinnamaldehyde	1,3-Dihydro-1-methyl-3-(2-oxopropylidene)-2H-indole-2-one	11	H08	Nordihydroguaiaretic acid from Larrea divaricata (creosote bush)	NDGA
03	H09	Cyclophosphamide monohydrate	2-[Bis(2-chloroethyl)amino]tetrahydr	11	H09	NADPH tetrasodium	Reduced nicotinamide adenosine dinucleotide phosphate tetrasodium

			o-2H-1,3,2-oxazaphosphorine 2-oxide				
03	H10	1-(4-Chlorobenzyl)-5-methoxy-2-methylindole-3-acetic acid		11	H10	Nilutamide	Anandron
03	H11	Cortisone	Kendall's Compound E; 4-Pregnene-17alpha,21-diol-3,11,20-trione; Reichstein's Substance Fa	11	H11	NO-711 hydrochloride	1-(2-(((Diphenylmethylene)imino)oxy)ethyl)-1,2,5,6-tetrahydro-3-pyridine-carboxylic acid hydrochloride
04	A02	Chelerythrine chloride	1,2-Dimethoxy-N-methyl(1,3)benzodioxolo(5,6-c)phenanthridinium chloride	12	A02	Nitrendipine	1,4-Dihydro-2,6-dimethyl-4-(3-nitrophenyl)-3,5-pyridinecarboxylic acid ethyl methyl ester
04	A03	Cyclosporin A	Antibiotic S 7481F1	12	A03	Naloxone benzoylhydrazone	[(5alpha)-4,5-Epoxy-3,14-dihydroxy-17-(2-propenyl)morphinan-6-ylidene]hydrazide benzoic acid
04	A04	Carbachol	Carbamylcholine chloride	12	A04	Olomoucine	2-[[9-Methyl-6-[(phenylmethyl)amino]-9H-purin-2-yl]amino]-ethanol
04	A05	Cephalexin hydrate		12	A05	Orphenadrine hydrochloride	beta-Dimethylaminoethyl 2-methylbenzhydryl ether hydrochloride
04	A06	Roscovitine	(R)-2-(1-Ethyl-2-hydroxyethylamino)-6-benzylamino-9-isopropylpurine	12	A06	(±)-Octoclothepin maleate	1-(8-Chloro-10,11-dihydrobenzo[b,f]thiepin-10-yl)-4-methyl-piperazine maleate
04	A07	Cyproheptadine hydrochloride		12	A07	O-Phospho-L-serine	L-Phosphoserine
04	A08	CB34	N,N-Dipropyl-2-(4-chlorophenyl)-6,8-dichloro-imidazo[1,2-a]pyridine-3-acetamide	12	A08	Pancuronium bromide	1,1'-([2beta,3alpha,5alpha,16beta,17beta]-3,17-Bis[acetyloxy]androstane-2,16-diyl)bis(1-methylpiperidinium) dibromide
04	A09	Cantharidin	Cantharidine	12	A09	Pentolinium di[L(+)-tartrate]	1,1'-Pentamethylenebis(1-methylpyrrolidinium hydrogen tartrate)
04	A10	Chlorpromazine hydrochloride		12	A10	Valproic acid sodium	2-Propylpentanoic acid sodium
04	A11	Centrophenoxine hydrochloride	Meclofenoxate hydrochloride	12	A11	Pyrilamine maleate	Mepyramine maleate
04	B02	1-(2-Chlorophenyl)-1-(4-chlorophenyl)-2,2-dichloroethane	Mitotane	12	B02	Nimodipine	1,4-Dihydro-2,6-dimethyl-4-(3-nitrophenyl)-3,5-pyridinecarboxylic acid 2-methoxyethyl 1-methylethyl ester
04	B03	D-Cycloserine	R(+)-4-Amino-3-isoxazolidinone	12	B03	NS-1619	1,3-Dihydro-1-[2-hydroxy-5-(trifluoromethyl)phenyl]-5-(trifluoromethyl)-2H-benzimidazol-2-one
04	B04	Chlorzoxazone	5-Chloro-2(3H)-benzoxazolone	12	B04	Oleic Acid	Elainic acid
04	B05	Chlorothiazide		12	B05	TG003	(Z)-1-(3-Ethyl-5-methoxy-2,3-dihydrobenzothiazol-2-ylidene)-propan-2-one
04	B06	SB 204741	N-(1-Methyl-1H-indo-5-yl)-N'-(3-methyl-5-isothiazolyl)urea	12	B06	Progesterone	4-Pregnene-3,20-dione

04	B07	GR 79236X	N-[(1S, trans)-2-Hydroxycyclopentyl]adenosine	12	B07	(±)-Propranolol hydrochloride	(±)-1-(Isopropylamino)-3-(1-naphthyloxy)-2-propanol hydrochloride
04	B08	Cefaclor		12	B08	3-alpha,21-Dihydroxy-5-alpha-pregnan-20-one	5-alpha-THDOC
04	B09	Citalopram hydrobromide	1-[3-(Dimethylamino)propyl]-1-(4-fluorophenyl)-1,3-dihydro-5-isobenzofurancarbonitrile hydrobromide	12	B09	1-Phenyl-3-(2-thiazolyl)-2-thiourea	
04	B10	Cefsulodin sodium salt hydrate	Sulcephalosporin	12	B10	Promethazine hydrochloride	
04	B11	Clemastine fumarate		12	B11	Piroxicam	4-Hydroxy-2-methyl-3-(pyrid-2-yl-carbamoyl)-2H-1,2-benzothiazine 1,1-dioxide
04	C02	(±)-Chlorpheniramine maleate		12	C02	Nisoxetine hydrochloride	LY-94,939
04	C03	8-(4-Chlorophenylthio)-cAMP sodium		12	C03	Naloxonazine dihydrochloride	Bis(5-alpha-4,5-epoxy-3,14,-dihydroxy-17-[2-propenyl]morphinan-6-ylidene)hydrazone
04	C04	L-Cysteinesulfinic Acid		12	C04	Oxymetazoline hydrochloride	3-[(4,5-Dihydro-1H-imidazol-2-yl)methyl]-6-(1,1-dimethylethyl)-2,4-dimethylphenol hydrochloride
04	C05	(+)-Chlorpheniramine maleate	(gammaS)-gamma-(4-Chlorophenyl)-N,N-dimethyl-2-pyridinepropanamine maleate	12	C05	Ofloxacin	Ofloxacine; DL-8280; HOE-280
04	C06	Ceftriaxone sodium	Ro-13-9904/001	12	C06	Palmitoylethanolamide	Palmidrol
04	C07	Cefmetazole sodium	CS-1170; SKF-83088	12	C07	SKF-525A hydrochloride	Proadifen hydrochloride
04	C08	DL-Cycloserine	4-amino-3-isoxazolidinone	12	C08	Pirfenidone	5-Methyl-1-phenyl-2-(1H)-pyridone
04	C09	Clonidine hydrochloride		12	C09	Thiolactomycin	[R-(E)]-4-hydroxy-3,5-dimethyl-5-(2-methyl-1,3-butadienyl)-2(5H)-thiophenone
04	C10	Caffeic acid phenethyl ester	CAPE	12	C10	Praziquantel	2-(Cyclohexylcarbonyl)-1,2,3,6,7-11b-hexahydro-4H-pyrazinoe(2,1a)isoquinolin-4-one
04	C11	beta-Chloro-L-alanine hydrochloride		12	C11	3-n-Propylxanthine	Enprofylline
04	D02	Cortisone 21-acetate	21-Acetoxy-4-pregnen-17alpha-ol-3,11,20-trione	12	D02	Nylidrin hydrochloride	
04	D03	Calmidazolium chloride	R 24571 chloride	12	D03	NBQX disodium	FG9202 disodium
04	D04	9-cyclopentyladenine	9-CP-Ade	12	D04	Sodium Oxamate	Oxalic acid monoamide sodium salt
04	D05	Cefazolin sodium	Sodium CEZ; SKF-41558	12	D05	Oxotremorine sesquifumarate salt	1-(4-[1-Pyrrolidinyl]-2-butynyl)-2-pyrrolidinone
04	D06	4-Chloromercuribenzoic acid	4-(Hydroxymercuri)benzoic acid	12	D06	Piceatannol	(E)-4-[2-(3,5-Dihydroxyphenyl)ethenyl]1,2-benzenediol
04	D07	Clozapine	8-Chloro-11-(4-methyl)-1-piperazinyl)-5H-dibenzo[b,e][1,4]diazepine	12	D07	Picrotoxin	
04	D08	McN-A-343	(4-Hydroxy-2-butynyl)-1-trimethylammonium-m-chlorocarbanilate chloride	12	D08	1,3-Dimethyl-8-phenylxanthine	8-Phenyltheophylline

04	D09	Cefotaxime sodium	Cefotaxim sodium	12	D09	Cisplatin	cis-Diammineplatinum(II) dichloride
04	D10	Cephapirin sodium	BL-P-1322	12	D10	Propafenone hydrochloride	1-(2-[2-Hydroxy-3-(propylamino)propoxy]phenyl)-3-phenyl-1propanone
04	D11	Pyrocatechol	1,2-Benzenediol; Catechol	12	D11	Phenylephrine hydrochloride	
04	E02	Cephalosporin C zinc salt		12	E02	SB 242084 dihydrochloride hydrate	6-Chloro-2,3-dihydro-5-methyl-N-[6-[(2-methyl-3-pyridinyl)oxy]-3-pyridinyl]-1H-indole-1-carboxyamide dihydrochloride hydrate
04	E03	GR 113808	1-Methyl-1H-indole-3-carboxylic acid, [1-[2-[(methylsulfonyl)amino]ethyl]-4-piperidinyl]methyl ester	12	E03	NS 2028	4H-8-Bromo-1,2,4-oxadiazolo(3,4-d)benz(b)(1,4)oxazin-1-one
04	E04	Cephalothin sodium		12	E04	Oxybutynin Chloride	alpha-Phenylcyclohexaneglycolic acid 4-(diethylamino)-2-butynyl ester hydrochloride
04	E05	Clemizole hydrochloride	1-p-Chlorobenzyl-2-(1-pyrrolidinyl)methylbenzimidazole hydrochloride	12	E05	TPCA-1	[5-(p-Fluorophenyl)-2-ureido]thiophene-3-carboxamide
04	E06	(-)-Cotinine	S(-)-1-Methyl-5-(3-pyridyl)-2-pyrrolidone	12	E06	Pentamidine isethionate	4'4-[1,5-Pentanediylbis(oxy)]bis-benzenecarboximidamide isethionate
04	E07	(±)-p-Chlorophenylalanine	p-CPA	12	E07	4-Phenyl-3-furoxancarbonitrile	Furoxan
04	E08	N-(2-[4-(4-Chlorophenyl)piperazin-1-yl]ethyl)-3-methoxybenzamide		12	E08	PRE-084	
04	E09	Cilostamide	OPC 3689	12	E09	Podophyllotoxin	
04	E10	Cephradine	Cefradin; SQ-11436	12	E10	5alpha-Pregnan-3alpha-ol-11,20-dione	Alfaxalone
04	E11	Z-L-Phe chloromethyl ketone	N-Carbobenzyloxy-L-phenylalanyl chloromethyl ketone; ZPCK	12	E11	Perphenazine	
04	F02	CGP-74514A hydrochloride	N2-(cis-2-Aminocyclohexyl)-N6-(3-chlorophenyl)-9-ethyl-9H-purine-2,6-diamine hydrochloride; Compound 13	12	F02	Naltriben methanesulfonate	NTB
04	F03	Carbamazepine	5H-Dibenz[b,f]azepine-5-carboxamide	12	F03	(±)-Octopamine hydrochloride	(±)-alpha-(Aminomethyl)-4-hydroxybenzyl alcohol hydrochloride
04	F04	Cimetidine	SKF-92334; Tagamet	12	F04	Oxiracetam	4-Hydroxy-2-oxopyrrolidine-N-acetamide
04	F05	2-Chloroadenosine	2-CADO	12	F05	SB 216763	3-(2,4-Dichlorophenyl)-4-(1-methyl-1H-indol-3-yl)-1H-pyrrole-2,5-dione
04	F06	CL 316,243	Disodium 5-[(2R)-2-[[(2R)-2-(3-Chlorophenyl)-2-hydroxyethyl]amino]propyl]-1,3-benzodioxole-2,2-dicarboxylate	12	F06	TBB	NSC 231634; 4,5,6,7-Tetrabromo-2-azabenzimidazole
04	F07	Chloroquine diphosphate		12	F07	Pentoxifylline	3,7-Dihydro-3,7-dimethyl-1-(5-oxohexyl)-1H-purine-2,6-dione
04	F08	Cystamine dihydrochloride	Decarboxycystine dihydrochloride	12	F08	PPNDS tetrasodium	Pyridoxal-5'-phosphate-6-(2'-naphthylazo-6'-nitro-4',8'-disulfonate) tetrasodium

04	F09	Chelidamic acid	4-Hydroxypyridine-2,6-dicarboxylic acid	12	F09	SU 9516	
04	F10	DSP-4 hydrochloride	N-(2-Chloroethyl)-N-ethyl-2-bromobenzylamine hydrochloride	12	F10	PNU-282987	N-(3R)-1-Azabicyclo[2.2.2]oct-3-yl-4-chloro-benzamide monohydrochloride
04	F11	CPCCOEt	7-(Hydroxyimino)cyclopropa[b]chromen-1a-carboxylate ethyl ester	12	F11	Pentylenetetrazole	Metrazole
04	G02	Cyproterone acetate	6-Chloro-1beta,2beta-dihydro-17-hydroxy-3'H-cyclopropa(1,2)-pregna-1,4,6-triene-3,20-dione acetate	12	G02	Naftopidil dihydrochloride	KT-611 dihydrochloride
04	G03	Captopril	(S)-1-(3-Mercapto-2-methyl-1-oxo-propyl)-L-proline	12	G03	N-Oleoylethanolamine	N-(Hydroxyethyl)oleamide
04	G04	Cyclobenzaprine hydrochloride	5-(3-Dimethylaminopropylidene) dibenzo[a,e]cycloheptatriene hydrochloride	12	G04	Ouabain	Acocantherine
04	G05	Bethanechol chloride	Carbamyl-beta-methylcholine chloride	12	G05	Oxaprozin	Daypro
04	G06	7-Chloro-4-hydroxy-2-phenyl-1,8-naphthyridine		12	G06	Parthenolide	
04	G07	Clofibrate		12	G07	Pimozide	
04	G08	Clomipramine hydrochloride	Anafranil hydrochloride; Chloripramine	12	G08	PD 404,182	6H-6-Imino-(2,3,4,5-tetrahydropyrimido)[1,2-c]-[1,3]benzothiazine
04	G09	N6-Cyclopentyladenosine	CPA	12	G09	Palmitoyl-DL-Carnitine chloride	
04	G10	Cinoxacin	1-Ethyl-1,4-dihydro-4-oxo[1,3]dioxolo[4,5-g]cinnoline-3-carboxylic acid	12	G10	Piracetam	2-Oxo-1-pyrrolidineacetamide
04	G11	Colchicine		12	G11	(+)-Pilocarpine hydrochloride	
04	H02	DL-p-Chlorophenylalanine methyl ester hydrochloride		12	H02	Bisoprolol hemifumarate salt	1-[4-[[2-(1-Methylethoxy)ethoxy]methyl] phenoxy]-3-[(1-methylethyl)amino]-2-propanol
04	H03	CNS-1102	N-(3-Ethylphenyl)-N-methyl-N′-1-naphthalenylguanidine monohydrochloride; Cerestat; Aptiganel hydrochloride	12	H03	Oxolinic acid	W-4565; 5,8-Dihydro-5-ethyl-8-oxo-1,3-dioxolo[4,5-g]quinoline-7-carboxylic acid
04	H04	Carbetapentane citrate	1-Phenyl-cyclopentanecarboxylic acid 2-[2-(Diethylamino)ethoxy]ethyl ester citrate	12	H04	ODQ	1H-[1,2,4]Oxadiazolo[4,3-a]quinoxalin-1-one
04	H05	Cinnarizine	1-trans-Cinnamyl-4-diphenylmethylpiperazine	12	H05	Oxotremorine methiodide	N,N,N-Trimethyl-4-(2-oxo-1-pyrrolidinyl)-2-butyn-1-ammonium iodide
04	H06	Clotrimazole	1-(o-Chlorotrityl)imidazole	12	H06	Pindolol	
04	H07	Cytosine-1-beta-D-arabinofuranoside hydrochloride	Arabinocytidine hydrochloride; Arabinosylcytosine hydrochloride; Ara-C hydrochloride	12	H07	L-Glutamic acid, N-phthaloyl-	

04	H08	Calcimycin	A23187; Calcium ionophore A23187	12	H08	Papaverine hydrochloride	
04	H09	Cantharidic Acid		12	H09	R(-)-N6-(2-Phenylisopropyl)adenosine	R(-)-PIA
04	H10	Carisoprodol		12	H10	Phosphomycin disodium	Fosfomycin; Phosphonomycin; MK-955
04	H11	L-Canavanine sulfate	L-alpha-Amino-gamma-(guanidinooxy)butyric acid sulfate	12	H11	Pilocarpine nitrate	
05	A02	Cyclothiazide	6-Chloro-3,4-dihydro-3-(2-norbornen-5-yl)-2H-1,2-4-benzothiadiazine-7-sulfonamide 1,1-dioxide	13	A02	Promazine hydrochloride	10-(3-[Dimethylamino]propyl)phenothiazine hydrochloride
05	A03	(±)-CPP	(±)-3-(2-Carboxypiperazin-4-yl)propyl-1-phosphonic acid	13	A03	Pirenzepine dihydrochloride	5,11-Dihydro-11-[(4-methyl-1-piperazinyl)acetyl]-6H-pyrido[2,3-b][1,4]benzodiazepin-6-one dihydrochloride
05	A04	CGS-21680 hydrochloride	2-p-(2-Carboxyethyl)phenethylamino-5'-N-ethylcarboxamidoadenosine hydrochloride	13	A04	1,3-PBIT dihydrobromide	Phenylene-1,3-bis(ethane-2-isothiourea) dihydrobromide
05	A05	CGS-15943	9-Chloro-2-(2-furyl)[1,2,4]triazolo[1,5-c]quinazolin-5-amine	13	A05	(±)-cis-Piperidine-2,3-dicarboxylic acid	
05	A06	Chloro-IB-MECA	2-Chloro-N6-(3-iodobenzyl)-adenosine-5'-N-methyluronamide	13	A06	Piribedil maleate	2-(4-[1,3-Benzodioxol-5-ylmethyl]-1-piperazinyl)pyrimidine
05	A07	Debrisoquin sulfate	3,4-Dihydro-2(1H)-isoquinolinecarboximidamide sulfate; Ro 5-33071	13	A07	Procaine hydrochloride	Novocaine hydrochloride
05	A08	Diltiazem hydrochloride		13	A08	Phaclofen	3-Amino-2-(4-chlorophenyl)propylphosphonic acid
05	A09	(S)-3,5-Dihydroxyphenylglycine	S-DHPG	13	A09	Pregnenolone sulfate sodium	5-Pregnen-3beta-ol-20-one sulfate sodium
05	A10	Phenytoin sodium	5,5-Diphenylhydantoin sodium	13	A10	PD 168,077 maleate	N-[[4-(2-Cyanophenyl)-1-piperazinyl]methyl]-3-methyl-benzamide maleate
05	A11	Daphnetin	7,8-Dihydroxycoumarin	13	A11	Quinacrine dihydrochloride	
05	B02	N6-Cyclohexyladenosine	CHA	13	B02	Phenelzine sulfate	
05	B03	CGS-12066A maleate	7-Trifluoromethyl-4(4-methyl-1-piperazinyl)-pyrrolo[1,2-a]quinoxaline maleate	13	B03	Putrescine dihydrochloride	Putrescine dihydrochloride
05	B04	Y-27632 dihydrochloride	(R)-(+)-trans-4-(1-Aminoethyl)-N-(4-Pyridyl)cyclohexanecarboxamide dihydrochloride	13	B04	Protoporphyrin IX disodium	Kammerer's porphyrin
05	B05	2-Chloro-2-deoxy-D-glucose	2-Chloro-DG	13	B05	Protriptyline hydrochloride	
05	B06	WB-4101 hydrochloride	2-(2,6-Dimethoxyphenoxyethyl)aminomethyl-1,4-benzodioxane hydrochloride	13	B06	Paromomycin sulfate	
05	B07	2',3'-didehydro-3'-deoxythymidine	2',3'-Anhydrothymidine; d4T	13	B07	2-Phenylaminoadenosine	CV-1808

05	B08	Dextromethorphan hydrobromide monohydrate	d-3-Methoxy-N-methylmorphinan hydrobromide	13	B08	BF-170 hydrochloride	2-(4-Aminophenyl)quinoline hydrochloride
05	B09	Dequalinium chloride hydrate	1,1'-Decamethylenebis(4-aminoquinaldinium) dichloride	13	B09	PPADS	Pyridoxal-phosphate-6-azophenyl-2',4'-disulphonic acid tetrasodium
05	B10	Doxepin hydrochloride		13	B10	SU 6656	2,3-Dihydro-N,N-dimethyl-2-oxo-3-[(4,5,6,7-tetrahydro-1H-indol-2-yl)methylene]-1H-indole-5-sulfonamide
05	B11	DM 235		13	B11	Quazinone	Ro 13-6438
05	C02	(S)-(+)-Camptothecin		13	C02	Pheniramine maleate	
05	C03	2-Cyclooctyl-2-hydroxyethylamine hydrochloride	CONH hydrochloride	13	C03	Phentolamine mesylate	Regitin mesylate
05	C04	1-(m-Chlorophenyl)-biguanide hydrochloride	m-CPBG hydrochloride	13	C04	1,4-PBIT dihydrobromide	1,4-Phenylene-bis(1,2-ethanediyl)bis-isothiourea
05	C05	Tocainide hydrochloride	2-Amino-N-(2,6-dimethylphenyl)propanamide hydrochloride	13	C05	Pergolide methanesulfonate	8-[(Methylthio)methyl]-6-propylergoline methanesulfonate
05	C06	DNQX	6,7-Dinitroquinoxaline-2,3-dione	13	C06	1,10-Phenanthroline monohydrate	o-Phenanthroline monohydrate
05	C07	Droperidol	1-[1-[3-(p-Fluorobenzoyl)propyl]-1,2,3,6-tetrahydro-4-pyridyl]-2-benzimidazolinone	13	C07	R(+)-3PPP hydrochloride	R(+)-3-(3-Hydroxyphenyl)-N-propylpiperidine hydrochloride
05	C08	Carvedilol	1-(9H-Carbazol-4-yloxy)-3-[[2-(2-methoxyphenoxy)ethyl]amino]-2-propanol	13	C08	1-Phenylbiguanide	N-Phenyl-imidocarbonimidic diamide
05	C09	Doxylamine succinate		13	C09	S(+)-PD 128,907 hydrochloride	S(+)-(4aR,10bR)-3,4,4a,10b-Tetrahydro-4-propyl-2H,5H-[1]benzopyrano-[4,3-b]-1,4-oxazin-9-ol hydrochloride
05	C10	S(-)-Pindolol		13	C10	Quinolinic acid	Pyridine-2,3-dicarboxylic acid
05	C11	5,5-Dimethyl-1-pyrroline-N-oxide	DMPO	13	C11	(-)-Quinpirole hydrochloride	LY-171,555
05	D02	CK2 Inhibitor 2	DMAT; 2-Dimethylamino-4,5,6,7-tetrabromo-1H-benzimidazole	13	D02	Phosphonoacetic acid	
05	D03	5-Carboxamidotryptamine maleate	5-CT; AH-21467	13	D03	Propionylpromazine hydrochloride	
05	D04	2-Chloroadenosine triphosphate tetrasodium	2-Chloro-ATP tetrasodium	13	D04	Phenylbutazone	
05	D05	Cirazoline hydrochloride	2-[(2-Cyclopropylphenoxy)methyl]-4,5-dihydro-1H-imidazole hydrochloride	13	D05	6(5H)-Phenanthridinone	
05	D06	Dihydroouabain		13	D06	Procainamide hydrochloride	
05	D07	L-3,4-Dihydroxyphenylalanine methyl ester hydrochloride	Methyl L-DOPA hydrochloride	13	D07	S(-)-3PPP hydrochloride	Preclamol hydrochloride
05	D08	Dihydroergotamine methanesulfonate		13	D08	SKF 94836	N-Cyano-N'-methyl-N''-[4-(1,4,5,6-tetrahydro-4-methyl-6-oxo-3-pyridazinyl)phenyl]guanidine
05	D09	Desipramine hydrochloride		13	D09	Phenamil methanesulfonate	3,5-Diamino-6-chloro-N-[imino(phenylamino)methyl]-pyrazinecarboxamide methansulfonate

05	D10	(-)-alpha-Methylnorepinephrine	Levonordefrin; (-)-3,4-Dihydroxynorephedrine	13	D10	Quercetin dihydrate	3,3',4',5,7-Pentahydroxyflavone dihydrate
05	D11	2',3'-dideoxycytidine	ddC	13	D11	Quipazine, N-methyl-, dimaleate	2-[1-(4-Methyl)-piperazinyl]quinoline dimaleate
05	E02	(+)-cis-Dioxolane	L(+)-cis-2-Methyl-4-trimethylammoniummethyl-1,3-dioxolane iodide	13	E02	(-)-Perillic acid	4-Isopropenyl-1-cyclohexene-1-carboxylic acid
05	E03	Cetirizine dihydrochloride	[2-[4-[(4-Chlorophenyl)phenylmethyl]-1-piperazinyl]ethoxy]acetic acid dihydrochloride	13	E03	Prazosin hydrochloride	
05	E04	(+)-Cyclazocine		13	E04	Picotamide	4-Methoxy-N,N'-bis(3-pyridinylmethyl)-1,3-benzenedicarboxamide
05	E05	CGP 20712A methanesulfonate	(+/-)-2-Hydroxy-5-[2-[[2-hydroxy-3-[4-[1-methyl-4-(trifluoromethyl)-1H-imidazol-2-yl]phenoxy]propyl]amino]ethoxy]-benzamide methanesulfonate	13	E05	5alpha-Pregnan-3alpha-ol-20-one	Allopregnan-3alpha-ol-20-one
05	E06	Dobutamine hydrochloride	(±)-4-[2-[[3-(4-Hydroxyphenyl)-1-methylpropyl]amino]ethyl]-1,2-benzenediol hydrochloride	13	E06	Prilocaine hydrochloride	N-(2-Methylphenyl)-2-(propylamino)propanamide hydrochloride
05	E07	1,4-Dideoxy-1,4-imino-D-arabinitol	DAB; 2-Hydroxymethyl-3,4-pyrrolidinediol	13	E07	(±)-PPHT hydrochloride	(±)-2-(N-Phenylethyl)-N-propyl)amino-5-hydroxytetralin hydrochloride
05	E08	Diphenyleneiodonium chloride	[1,1'-Biphenyl]-2,2'-diyiodonium chloride	13	E08	Pirenperone	R-47,465
05	E09	N-Methyl-1-deoxynojirimycin	1,5-Dideoxy-1,5-imino-1-methyl-D-sorbitol	13	E09	Phenylbenzene-omega-phosphono-alpha-amino acid	PMBA
05	E10	Dilazep hydrochloride	Cormelian	13	E10	Quinidine sulfate	
05	E11	Diacylglycerol Kinase Inhibitor II	R59949	13	E11	Quipazine, 6-nitro-, maleate	6-Nitro-2-(1-piperzinyl)-quinoline maleate
05	F02	OXA-22	cis-2-Methyl-5-trimethylammoniummethyl-1,3-oxathiolane iodide	13	F02	Pyrazinecarboxamide	Pyrazinoic acid amide; pyrazinamide; PZA
05	F03	(±)-CGP-12177A hydrochloride	4-[4-[(1,1-Dimethylethyl)amino]-2-hydroxypropoxy]-1,3-dihydro-2H-benzimidazol-2-one hydrochloride	13	F03	Phloretin	3-(4-Hydroxyphenyl)-1-(2,4,6-trihydroxyphenyl)-1-propanone
05	F04	Capsazepine	N-[2-(4-Chlorophenyl)ethyl]-1,3,4,5-tetrahydro-7,8-dihydroxy-2H-2-benzazepine-2-carbothioamide	13	F04	Tranylcypromine hydrochloride	(±)-trans-2-Phenylcyclopropylamine hydrochloride
05	F05	(2S,1'S,2'S)-2-(carboxycyclopropyl)glycine	L-CCG-1	13	F05	Propantheline bromide	(2-Hydroxyethyl)diisopropylmethylammonium bromide xanthene-9-carboxylate bromide
05	F06	Dihydrokainic acid	2-Carboxy-4-isopropyl-3-pyrrolidineacetic acid	13	F06	Propentofylline	HWA 285
05	F07	2,4-Dinitrophenyl 2-fluoro-2-deoxy-beta-D-glucopyranoside		13	F07	Org 27569	5-chloro-3-ethyl-1H-indole-2-carboxylic acid [2-(4-piperidin-1-yl-phenyl)-ethyl]-amide

05	F08	Diphenhydramine hydrochloride		13	F08	IC 261	1,3-Dihydro-3-[(2,4,6-trimethoxyphenyl)methylene]-2H-indol-2-one
05	F09	5,5-Diphenylhydantoin	Phenytoin	13	F09	Bay 11-7082	(E)-3-(4-Methylphenylsulfonyl)-2-propenenitrile
05	F10	Ganaxolone	3a-Hydroxy-3beta-methyl-5a-pregnan-20-one	13	F10	Quipazine dimaleate	2-(1-Piperazinyl)quinoline dimaleate
05	F11	Tyrphostin AG 1296	6,7-Dimethoxy-2-phenylquinoxaline	13	F11	Quinelorane dihydrochloride	LY-163,502
05	G02	8-Cyclopentyl-1,3-dipropylxanthine	DPCPX; PD 116,948	13	G02	Primidone	
05	G03	S-(-)-Carbidopa		13	G03	Pargyline hydrochloride	N-Methyl-N-2-propynylbenzylamine hydrochloride
05	G04	Chlormezanone	2-(4-Chlorophenyl)tetrahydro-3-methyl-4H-1,3-thiazin-4-one 1,1-dioxide	13	G04	(S)-Propranolol hydrochloride	(S)-1-Isopropylamino-3-(1-naphthyloxy)-2-propanol hydrochloride
05	G05	CNQX disodium	6-Cyano-7-nitroquinoxaline-2,3-dione	13	G05	K114	trans,trans)-1-Bromo-2,5-bis-(4-hydroxy)styrylbenzene
05	G06	Decamethonium dibromide	Decamethylene bis(trimethylammonium bromide)	13	G06	(S)-(-)-propafenone hydrochloride	(S)-1-(2-[2-Hydroxy-3-(propylamino)propoxy]phenyl)-3-phenyl-1-propanone hydrochloride
05	G07	D-ribofuranosylbenzimidazole	DRB; 5,6-Dichlorobenzimidazole riboside	13	G07	N6-2-Phenylethyladenosine	
05	G08	2,3-Butanedione	Biacetyl; BDM	13	G08	A3 hydrochloride	N-(2-Aminoethyl)-5-chloronaphthalene-1-sulfonamide hydrochloride
05	G09	N^G,N^G-Dimethylarginine hydrochloride	asym-Dimethylarginine hydrochloride ADMA	13	G09	PD 98,059	2-(2-Amino-3-methoxyphenyl)-4H-1-benzopyran-4-one
05	G10	1,7-Dimethylxanthine	Paraxanthine	13	G10	Quinine sulfate	
05	G11	N-Methyldopamine hydrochloride	Epinine hydrochloride; Deoxyepinephrine hydrochloride	13	G11	(±)-Quinpirole dihydrochloride	LY-141,865
05	H02	8-Cyclopentyl-1,3-dimethylxanthine	CPT; 8-Cyclopentyltheophylline	13	H02	(±)-threo-1-Phenyl-2-decanoylamino-3-morpholino-1-propanol hydrochloride	PDMP hydrochloride
05	H03	(±)-Chloro-APB hydrobromide	(±)-SKF-82958 hydrobromide	13	H03	Phorbol 12-myristate 13-acetate	PMA
05	H04	8-(3-Chlorostyryl)caffeine	CSC	13	H04	Ammonium pyrrolidinedithiocarbamate	APDC
05	H05	CX 546	1-(1,4-Benzodioxan-6-ylcarbonyl)piperidine	13	H05	Prochlorperazine dimaleate	
05	H06	P1,P4-Di(adenosine-5')tetraphosphate triammonium	Ap4A	13	H06	Pyridostigmine bromide	
05	H07	SANT-1	(4-Benzyl-piperazin-1-yl)-(3,5-dimethyl-1-phenyl-1H-pyrazol-4-ylmethylene)-amine	13	H07	N6-Phenyladenosine	
05	H08	N,N,N',N'-Tetramethylazodicarboxamide	Azodicarboxylic acid bis(dimethylamide); diamide	13	H08	Pinacidil	(±)-N-Cyano-N'-4-pyridinyl-N''-(1,2,2-trimethylpropyl)-guanidine
05	H09	Clodronic acid	Cl2MDP; Clodronic acid disodium magnesium salt; DMDP	13	H09	(±)-PD 128,907 hydrochloride	PD 125,530

05	H10	2,3-Dimethoxy-1,4-naphthoquinone	DMNQ	13	H10	(+)-Quisqualic acid	L(+)-alpha-Amino-3,5-dioxo-1,2,4-oxadiazolidine-2-propanoic acid
05	H11	1,1-Dimethyl-4-phenyl-piperazinium iodide	DMPP	13	H11	Cortexolone	11-Deoxycortisol
06	A02	PD 169316	4-(4-Fluorophenyl)-2-(4-nitrophenyl)-5-(4-pyridyl)-1H-imidazole	14	A02	Ritodrine hydrochloride	N-(p-Hydroxyphenethyl)-4-hydroxynorephedrine hydrochloride
06	A03	Disopyramide	alpha-Diisopropylaminoethyl-alpha-phenylpyridine-2-acetamide	14	A03	REV 5901	alpha-Pentyl-3-[2-quinolinylmethoxy]benzyl alcohol
06	A04	Dephostatin	2-(N-methyl-N-nitroso)hydroquinone	14	A04	Ro 8-4304	(4-[3-(4-[4-fluorophenyl]-3,6-dihydro-2H-pyridin-1-yl)-2-hydroxypropoxy]benzamide
06	A05	Diazoxide		14	A05	Ro 41-0960	2'-Fluoro-3,4-dihydroxy-5-nitrobenzophenone
06	A06	Doxycycline hydrochloride		14	A06	Ro 04-6790 dihydrochloride	4-Amino-N-[2,6-bis(methylamino)-4-pyrimidinyl]-benzenesulfonamide dihydrochloride
06	A07	R(-)-N-Allylnorapomorphine hydrobromide		14	A07	ST-148	N-(4-[4-(2-methoxyphenyl)-piperazin-1-yl]-butyl-5-(dimethylamino)-napthalene-1-sulfonamide maleate
06	A08	4-DAMP methiodide	4-Diphenylacetoxy-N-methylpiperidine methiodide	14	A08	Spermidine trihydrochloride	N-(3-Aminopropyl)-1,4-butanediamine trihydrochloride
06	A09	N,N-Dipropyl-5-carboxamidotryptamine maleate		14	A09	SB 204070 hydrochloride	1-Butyl-4-piperidinylmethyl-8-amino-7-chloro-2,3-dihydro-1,4-benzodioxin-5-carboxylate hydrochloride
06	A10	Dihydroergocristine methanesulfonate		14	A10	IRAK-1/4 Inhibitor I	1-(2-(4-Morpholinyl)ethyl)-2-(3-nitrobenzoylamino)benzimidazole
06	A11	Enoximone	1,3-Dihydro-4-methyl-5-[4-methylthiobenzoyl]-2H-imidazol-2-one	14	A11	(±)-Sulpiride	(±)-5-(Aminosulfonyl)-N-[(1-ethyl-2-pyrrolidinyl)methyl]-2-methoxybenzamide
06	B02	Disopyramide phosphate		14	B02	Raloxifene hydrochloride	LY 139481
06	B03	Daidzein	7-Hydroxy-3-(4-hydroxyphenyl)-4H-1-benzopyran-4-one	14	B03	Rottlerin	Mallotoxin
06	B04	3',4'-Dichlorobenzamil	L-594,881	14	B04	RX 821002 hydrochloride	2-[2-(2-Methoxy-1,4-benzodioanyl)]-imidazoline hydrochloride
06	B05	3,4-Dihydroxyphenylacetic acid	DOPAC	14	B05	Reactive Blue 2	Basilen blue E-3G
06	B06	6,7-ADTN hydrobromide	(±)-2-Amino-6,7-dihydroxy-1,2,3,4-tetrahydro-naphthalene hydrobromide	14	B06	(±)-Sotalol hydrochloride	N-(4-[1-Hydroxy-2-(isopropylamino)ethyl]phenyl) methanesulfonamide hydrochloride
06	B07	Mephetyl tetrazole	1-[2-(4-Methoxyphenyl)ethyl]-5-(1-p-tolylcyclopropyl)-1H-tetrazole	14	B07	SKF 86466	6-Chloro-2,3,4,5-tetrahydro-3-methyl-1H-3-benzazepine hydrochloride
06	B08	1,3-Dipropyl-7-methylxanthine		14	B08	SNC80	(+)-4-[(alphaR)-alpha-((2S,5R)-4-Allyl-2,5-dimethyl-1-piperazinyl)-3-methoxybenzyl]-N,N-diethylbenzamide

06	B09	6,7-Dichloroquinoxaline-2,3-dione	DCQX	14	B09	N-Oleoyldopamine	OLDA
06	B10	2,4-Diamino-6-pyrimidinone	DAHP; 2,4-Diamino-6-hydroxypyrimidine	14	B10	SB 269970 hydrochloride	[R]-3-[2-(2-[4-Methyl-piperidin-1-yl]ethyl)pyrrolidine-1-sulfonyl]phenol hydrochloride
06	B11	Etoposide	Lastet	14	B11	CV-3988	(+/-)-(3-(N-Octadecylcarbamoyloxy)-2-methoxy)propyl-2-thiazolioethyl phosphate
06	C02	Demeclocycline hydrochloride	7-chloro-6-demethyltetracycline hydrochloride	14	C02	Retinoic acid	Vitamin A acid
06	C03	Cilnidipine	1,4-Dihydro-2,6-dimethyl-4-(3-nitrophenyl)-3,5-pyridinedicarboxylic acid 2-methoxyethyl (2E)-3-phenyl-2-propenyl ester	14	C03	Ranolazine dihydrochloride	N-(2,6-Dimethylphenyl)-4-[2-hydroxy-3-(2-methoxyphenoxy) propyl]-1-piperazineacetamide dihydrochloride; (±) -4-[2-hydroxy-3-(o-methoxyphenoxy)propyl]-1-piperazineaceto-2',6'-xylidide dihydrochloride
06	C04	3-deazaadenosine	4-Amino-1-(beta-D-ribofuranosyl)-1H-imidazo(4,5)-pyridine	14	C04	Ribavirin	1-beta-D-Ribofuranosyl-1,2,4-triazole-3-carboxamide
06	C05	Dantrolene sodium	1-([5-(p-Nitrophenyl)furfurylidene]amino)hydantoin	14	C05	Riluzole	2-Amino-6-(trifluoromethoxy)-benzothiazole
06	C06	R(-)-Apocodeine hydrochloride	R(-)-10-Methoxy-11-hydroxyaporphine hydrochloride	14	C06	SB-366791	Vanilloid receptor-1 antagonist
06	C07	Icilin	AG-3-5; 1-(2-Hydroxyphenyl)-4-(3-nitrophenyl)-1,2,3,6-tetrahydropyrimidin-2-one	14	C07	SR 57227A	4-amino-1-(6-chloro-2-pyridyl)-piperidine hydrochloride
06	C08	Domperidone		14	C08	SKF 83959 hydrobromide	6-chloro-7,8-dihydroxy-3-methyl-1-(3-methylphenyl)-2,3,4,5-tetrahydro-1H-3-benzazepine hydrobromide
06	C09	3,7-Dimethyl-1-propargylxanthine	DMPX	14	C09	Spironolactone	
06	C10	DL-alpha-Difluoromethylornithine hydrochloride	DFMO hydrochloride	14	C10	Spiperone hydrochloride	R 5147 hydrochloride; Spiroperidol hydrochloride
06	C11	ET-18-OCH3	3,5,9-Trioxa-4-phosphaheptacosan-1-aminium	14	C11	Sulindac	(Z)-5-Fluoro-2-methyl-1-[[4-(methyl-sulfinyl)phenyl]methylene]-1H-indene-3-acetic acid
06	D02	Diethylenetriaminepentaacetic acid	Pentetic acid; DTPA	14	D02	Ruthenium red	Ammoniated ruthenium oxychloride
06	D03	Dicyclomine hydrochloride	2-(Diethylamino)ethyl 1-cyclohexylcyclohexane-1-carboxylate hydrochloride	14	D03	Rolipram	ZK 62711
06	D04	Cytidine 5'-diphosphocholine sodium salt hydrate	Citicholine sodium	14	D04	Ranitidine hydrochloride	Zantac
06	D05	DCEBIO	5,6-Dichloro-1-ethyl-1,3-dihydro- 2H-benzimidazol-2-one	14	D05	Steviol	(4alpha)-13-Hydroxykaur-16-en-18-oic acid
06	D06	R(-)-Propylnorapomorphine hydrochloride	R(-)-NPA hydrochloride	14	D06	Sodium nitroprusside dihydrate	Sodium nitroferricyanide

06	D07	(±)-SKF-38393 hydrochloride	(±)-1-Phenyl-2,3,4,5-tetrahydro-(1H)-3-benzazepine-7,8-diol hydrochloride		14	D07	(-)-Scopolamine hydrobromide	Hyoscine hydrobromide	
06	D08	Propofol			14	D08	Spermine tetrahydrochloride	N,N'-bis(3-Aminopropyl)-1,4-butanediamine tetrahydrochloride	
06	D09	5,7-Dichlorokynurenic acid	5,7-Dichloro-4-hydroxyquinoline-2-carboxylic acid		14	D09	SCH-202676 hydrobromide	N-(2,3-diphenyl-1,2,4-thiadiazol-5-(2H)-ylidene)methanamine hydrobromide	
06	D10	SCH-28080	2-Methyl-8-(phenylmethoxy)imidazo[1,2-a]pyridine-3-acetonitrile		14	D10	SR 2640	2-[[3-(2-Quinolinylmethoxy)phenyl]amino]-benzoic acid; QMPB	
06	D11	Etazolate hydrochloride	SQ 20,009; 1-Ethyl-4-[(1-methylethylidene)hydrazino]-1H-pyrazolo[3,4-b]pyridine-5-carboxylic acid ethyl ester hydrochloride		14	D11	Succinylcholine chloride		
06	E02	Diclofenac sodium	2-[(2,6-Dichlorophenyl)amino]benzeneacetic acid sodium		14	E02	13-cis-retinoic acid	Isotretinoin	
06	E03	3,4-Dichloroisocoumarin	3,4-DCI		14	E03	Ro 25-6981 hydrochloride	(R-[R*,S*])-alpha-(4-Hydroxyphenyl)-beta-methyl-4-(phenylmethyl)-1-peperidinepropanol) hydrochloride	
06	E04	Danazol			14	E04	Ritanserin	6-[2-[4-bis(4-Fluorophenyl)methylene]-1-piperidinyl]-ethyl]-7-methyl-5H-thiazolo[3,2-a]pyrimidin-5-one	
06	E05	1-Deoxynojirimycin hydrochloride	DNM; 1,5-Dideoxy-1,5-imino-D-sorbitol hydrochloride		14	E05	S(+)-Raclopride L-tartrate		
06	E06	R(-)-2,10,11-Trihydroxyaporphine hybrobromide	R(-)-2-Hydroxyapomorphine hydrobromide		14	E06	(±)-Synephrine	4-hydroxy-alpha-(methylaminomethyl)benzyl alcohol	
06	E07	GBR-12909 dihydrochloride	1-[2-[bis(4-Fluorophenyl)methoxy]ethyl]-4-[3-phenylpropyl]piperazine dihydrochloride		14	E07	SC-560	5-(4-Chlorophenyl)-1-(4-methoxyphenyl)-3-trifluoromethyl pyrazole	
06	E08	Nefiracetam	2-(2-Oxopyrrolidin-1-yl)-N-(2,6-dimethylphenyl)-acetamide		14	E08	SKF 75670 hydrobromide	7,8-dihydroxy-3-methyl-1-phenyl-2,3,4,5-tetrahydro-1H-3-benzazepine hydrobromide	
06	E09	4-Diphenylacetoxy-N-(2-chloroethyl)piperidine hydrochloride	4-DAMP mustard hydrochloride		14	E09	D-Serine	R(-)-2-Amino-3-hydroxypropionic acid	
06	E10	Venlafaxine hydrochloride	(+/-)-1-[2-(Dimethylamino)-1-(4-methoxyphenyl)ethyl]cyclohexanol hydrochloride		14	E10	(-)-Sulpiride	(-)-5-Aminosulfonyl-N-[(1-ethyl-2-pyrrolidinyl)methyl]-2-methoxybenzamide	
06	E11	7-Cyclopentyl-5-(4-phenoxy)phenyl-7H-pyrrolo[2,3-d]pyrimidin-4-ylamine			14	E11	Salbutamol	Albuterol	
06	F02	DL-erythro-Dihydrosphingosine	DL-Sphinganine		14	F02	Rutaecarpine	Rutecarpine	
06	F03	DBO-83	3-(6-Chloro-3-pyridazinyl)-3,8-diazabicyclo[3.2.1]octane dihydrochloride		14	F03	Phosphoramidon disodium	N-(alpha-Rhamnopyranosyloxyhydroxyphosphinyl)-Leu-Trp disodium	

06	F04	N,N-Dihexyl-2-(4-fluorophenyl)indole-3-acetamide	FGIN-1-27	14	F04	Rauwolscine hydrochloride	alpha-Yohimbine hydrochloride
06	F05	L-3,4-Dihydroxyphenylalanine	L-DOPA; Levodopa	14	F05	Sobuzoxane	4,4'-(1,2-Ethanediyl)bis(1-isobutoxycarbonyloxymethyl-2,6-piperazinedione)
06	F06	R(-)-2,10,11-Trihydroxy-N-propylnoraporphine hydrobromide	R(-)-TNPA HBr; R(-)-2-OH-NPA hydrobromide	14	F06	Sulfaphenazole	4-Amino-N-(1-phenyl-1H-pyrazol-5-yl)-benzenesulfonamide
06	F07	R(+)-SCH-23390 hydrochloride	R(+)-7-Chloro-8-hydroxy-3-methyl-1-phenyl-2,3,4,5-tetrahydro-1H-3-benzazepine hydrochloride	14	F07	Semicarbazide hydrochloride	Hydrazine carboxamide hydrochloride
06	F08	R(+)-Butylindazone	R(+)-DIOA	14	F08	SC 19220	2-acetylhydrazide 10(11H)-carboxylic acid
06	F09	1,10-Diaminodecane	DA10; Decamethylenediamine	14	F09	Albuterol hemisulfate	Salbutamol hemisulfate
06	F10	Vanillic acid diethylamide	Ethamivan	14	F10	SKF 96365	1-(beta-[3-(4-Methoxyphenyl)propoxy]-4-methoxyphenethyl)-1H-imidazole hydrochloride
06	F11	Emetine dihydrochloride hydrate		14	F11	Salmeterol xinafoate	(±) 4-Hydroxy-a1-[[[6-(4-phenylbutoxy)hexyl]amino]m-ethyl]-1,3-benzenedimethanol xinafoate; GR 33343X xinafoate
06	G02	R-(-)-Desmethyldeprenyl hydrochloride	L-Nordeprenyl hydrochloride	14	G02	Ropinirole hydrochloride	SKF 101468; 4-[2-(dipropylamino)ethyl]-1,3-dihydro-2H-indol-2-one hydrochloride
06	G03	7,7-Dimethyl-(5Z,8Z)-eicosadienoic acid	DEDA	14	G03	BIA 2-093	S-(-)-10-Acetoxy-10,11-dihydro-5H-dibenz[b,f]azepine-5-carboxamide
06	G04	(R,R)-cis-Diethyl tetrahydro-2,8-chrysenediol	(5R, 11R)-5,11-Diethyl-5,6,11,12-tetrahydro-2,8-chrysenediol	14	G04	Ibandronate sodium	(1-Hydroxy-3-(methylpentylamino)propylidene)bisphosphonic acid sodium
06	G05	Dipyridamole		14	G05	Rilmenidine hemifumarate	N-(Dicyclopropylmethyl)-4,5-dihydro-2-oxazolamine; Oxaminozoline
06	G06	Dipropyldopamine hydrobromide		14	G06	PQ401	IGF-1R Inhibitor II; N-(5-Chloro-2-methoxyphenyl)-N'-(2-methylquinolin-4-yl)urea
06	G07	(±)-DOI hydrochloride	(±)-2,5-Dimethoxy-4-iodoamphetamine hydrochloride	14	G07	(-)-Scopolamine methyl nitrate	Hyoscine methyl nitrate
06	G08	Eliprodil	alpha-(4-Chlorophenyl)-4-[(4-fluorophenyl)methyl]-1-piperidineethanol	14	G08	SKF 89626	4-(3,4-dihydroxyphenyl)-4,5,6,7-tetrahydrothieno[2,3-c]pyridine
06	G09	Dihydro-beta-erythroidine hydrobromide	3beta-1,6-Didehydro-14,17-dihydro-3-methoxy-16(15H)-oxaerythrinan-15-one hydrobromide	14	G09	Sanguinarine chloride	13-Methyl-[1,3]benzodioxolo[5,6-c]-1,3-dioxolo[4,5-i] phenanthridinium chloride
06	G10	Epibestatin hydrochloride	([2R,3R]-3-Amino-2-hydroxy-4-phenylbutanoyl)-L-leucine hydrochloride	14	G10	(-)-Scopolamine,n-Butyl-, bromide	Butylscopolamine bromide
06	G11	5'-N-Ethylcarboxamidoadenosine	NECA	14	G11	SU 5416	1,3-Dihydro-3-[(3,5-dimethyl-1H-pyrrol-2-yl)methylene]-2H-indol-2-one
06	H02	2,2'-Bipyridyl	alpha,alpha'-Bipyridyl	14	H02	Resveratrol	5-[(1E)-2-(4-Hydroxyphenyl)ethenyl]-1,3-benzenediol

06	H03	(±) trans-U-50488 methanesulfonate	trans-(±)-3,4-Dichloro-N-methyl-N-[2-(1-pyrrolidinyl)-cyclohexyl]-benzeneacetamide methanesulfonate	14	H03	Rotenone	
06	H04	SP600125	Anthrapyrazolone; 1,9-Pyrazoloanthrone	14	H04	Ro 41-1049 hydrochloride	N-(2-Aminoethyl)-5-(3-fluorophenyl)-4-thiazolecarboxamide hydrochloride
06	H05	Doxazosin mesylate	1-(4-amino-6,7-dimethoxy-2-quinazolinyl)-4-[4-(1,4-benzodioxan-2-yl)carpiperazin-1-yl)]-6,7-dimethoxyquinazoline mesylate	14	H05	R(-)-Denopamine	(-)-alpha-(3,4-dimethoxyphenethylaminomethyl)-4-hydroxybenzylalcohol
06	H06	AC-93253 iodide	2-[3-(1,3-Dihydro-1,3,3-trimethyl-2H-indol-2-ylidene)-1-propenyl]-3-ethyl-benzothiazolium iodide	14	H06	Sulindac sulfone	(Z)-5-Fluoro-2-methyl-1-[p-(methylsulfonyl)benzylidene]indene-3-acetic acid
06	H07	(±)-2,3-Dichloro-alpha-methylbenzylamine hydrochloride	DCMB hydrochloride; LY-78335	14	H07	DL-Stearoylcarnitine chloride	
06	H08	3,5-Dinitrocatechol	OR-486	14	H08	SKF 83565 hydrobromide	6-chloro-1-(3-chlorophenyl)-7,8-dihydroxy-3-methyl-2,3,4,5-tetrahydro-1H-3-benzazepine hydrobromide
06	H09	AL-8810	(5Z, 13E)-(9S,11S,15R)-9,15,dihydroxy-11-fluoro-15-(2-indanyl)-16,17,18,19,20,pentanor-5,13-prostadienoic acid	14	H09	N-Succinyl-L-proline	
06	H10	Etodolac	1,8-Diethyl-1,3,4,9-tetrahydropyrano[3,4-b]indole-1-acetic acid	14	H10	SB 205384	4-Amino-7-hydroxy-2-methyl-5,6,7,8-tetrahydrobenzo[b]thieno[2,3-b]pyridine-3-carboxylic acid but-2-ynyl ester
06	H11	E-64	L-trans-3-Carboxyoxiran-2-carbonyl-L-leucylagmatine	14	H11	(-)-Scopolamine methyl bromide	Hyoscine methyl bromide
07	A02	SB 415286	3-[(3-Chloro-4-hydroxyphenyl)amino]-4-(2-nitrophenyl)-1H-pyrrol-2,5-dione	15	A02	SU 4312	3-(4-Dimethylaminobenzylidenyl)-2-indolinone
07	A03	rac-2-Ethoxy-3-octadecanamido-1-propylphosphocholine		15	A03	1-(2-Methoxyphenyl)piperazine hydrochloride	2-MPP hydrochloride
07	A04	(-)-Physostigmine	Eserine	15	A04	Sepiapterin	S(-)-2-Amino-7,8-dihydro-6-(2-hydroxy-1-oxopropyl)-4(1H)-pteridione
07	A05	S-(-)-Eticlopride hydrochloride	FLB 131	15	A05	Tiapride hydrochloride	N-(2-[Diethylamino]ethyl)-5-(methylsulfonyl)-o-anisamide hydrochloride
07	A06	UCL 2077	(3-Triphenylmethylaminomethyl)pyridine	15	A06	Trihexyphenidyl hydrochloride	
07	A07	Fenofibrate	2-[4-(4-Chlorobenzoyl)phenoxy]-2-methylpropanoic acid 1-methyl-ethyl ester	15	A07	Terbutaline hemisulfate	2-t-Butylamino-1-(3,5-dihydroxyphenyl)ethanol
07	A08	Forskolin		15	A08	Tyrphostin AG 1478	N-(3-Chlorophenyl)-6,7-dimethoxy-4-quinazolinamine
07	A09	Fexofenadine hydrochloride	Terfenidine carboxylate, MDL 16455	15	A09	Tyrphostin AG 528	N-(3',4'-Dihydroxybenzylidenecyanoacetyl)-indoline

07	A10	N-(3,3-Diphenylpropyl)glycinamide	N20C	15	A10	(±)-alpha-Lipoic Acid	(±)-1,2-Dithiolane-3-pentanoic acid
07	A11	L-Canavanine	L-alpha-Amino-gamma-(guanidinooxy)-n-butyric acid	15	A11	Triprolidine hydrochloride	(E)-2-[3-(1-Pyrrolidinyl)-1-p-tolylpropenyl]pyridine hydrochloride
07	B02	S-Ethylisothiourea hydrobromide	2-Ethyl-2-thiopseudourea hydrobromide	15	B02	SR 59230A oxalate	3-(2-ethylphenoxy)-1[(1S)-1,2,3,4-tertahydronaphth-1-ylamino]-(2S)-2-propanol oxalate
07	B03	N-Ethylmaleimide	NEM	15	B03	PAPP	LY-165,163; p-Aminophenethyl-m-trifluoromethylphenyl piperazine
07	B04	NBI 27914	5-Chloro-4-(N-(cyclopropyl)methyl-N-propylamino)-2-methyl-6-(2,4,6-trichlorophenyl)-aminopyridine	15	B04	R(-)-SCH-12679 maleate	R(-)-1-Phenyl-2,3,4,5-tetrahydro-1H-7,8-dimethoxy-3-benzazepine maleate
07	B05	CCG-4986	Methyl N-[(4-chlorophenyl)sulfonyl]-4-nitrobenzenesulfinimidoate	15	B05	Taurine	2-Aminoethanesulfonic acid
07	B06	Fluvoxamine maleate	(E)-5-Methoxy-1-[4-(trifluoromethyl)phenyl]-1-pentanone-O-(2-aminoethyl)oxime maleate	15	B06	Theophylline	1,3-Dimethylxanthine
07	B07	Fenspiride hydrochloride	8-(2-Phenylethyl)-1-oxa-3,8-diazaspiro[4.5]decan-2-one hydrochloride	15	B07	4-Hydroxyphenethylamine hydrochloride	Tyramine hydrochloride
07	B08	Famotidine	N'-(Aminosulfonyl)-3-([2-(diaminomethyleneamino)-4-thiazolyl]methylthio)propanamidine	15	B08	Tetrahydrozoline hydrochloride	
07	B09	Formoterol	(R*,R*)-N-[2-hydroxy-5-[1-hydroxy-2-[[2-(4-methoxyphenyl)-1-methylethyl]amino]ethyl]phenyl]formamide	15	B09	Terazosin hydrochloride	1-(4-Amino-6,7-dimethoxy-2-quinazolinyl)-4-[(tetrahydro-2-furanyl)carbonyl]piperazine hydrochloride
07	B10	Glybenclamide	Glyburide	15	B10	DL-Thiorphan	DL-3-Mercapto-2-benzylpropanoylglycine
07	B11	GW1929	N-(2-Benzoylphenyl)-O-[2-(methyl-2-pyridinylamino)ethyl]-L-tyrosine;N-(2-Benzoyl phenyl)-L-tyrosine	15	B11	Tyrphostin AG 112	3-Amino-2,4-dicyano-5-(4'-hydroxyphenyl)-penta-2,4-dienonitrile
07	C02	Epinastine hydrochloride	9,13b-Dihydro-1H-dibenz[cf]imidazo[1,5-a]azepine hydrochloride	15	C02	BRL 52537 hydrochloride	(+/-)-1-(3,4-Dichlorophenyl)acetyl-2-(1-pyrrolidinyl)methylpiperidine hydrochloride
07	C03	(-)-Epinephrine bitartrate	Adrenaline bitartrate	15	C03	Spiroxatrine	R 5188
07	C04	beta-Estradiol	Dihydrofolliculin	15	C04	(±)-SKF 38393, N-allyl-, hydrobromide	(±)-7,8-Dihydroxy-3-allyl-1-phenyl-2,3,4,5-tetrahydro-1H-3-benzazepine hydrobromide
07	C05	erythro-9-(2-Hydroxy-3-nonyl)adenine hydrochloride	EHNA hydrochloride	15	C05	Thiothixene hydrochloride	SKF-5019 hydrochloride
07	C06	Picropodophyllotoxin	(5R,5aS,8aR,9R)-9-Hydroxy-5,8,8a,9-tetrahydro-5-(3,4,5-trimethoxyphenyl)furo[3',4':6,7]naphtho[2,3-d]-1,3-dioxol-6(5aH)-one	15	C06	(E)-4-amino-2-butenoic acid	TACA

07	C07	Flumazenil	Ro 15-1788	15	C07	Triflupromazine hydrochloride	
07	C08	FSCPX	8-Cyclopentyl-N3-[3-(4-(fluorosulfonyl)benzoyloxy) propyl]-N1-propylxanthine	15	C08	Tyrphostin AG 494	N-Phenyl-3,4-dihydroxybenzylidenecyanoac etamide
07	C09	Felodipine	Plendil	15	C09	Tyrphostin AG 537	Bis-Tyrphostin
07	C10	GW2974	N4-(1-Benzyl-1H-indazol-5-yl)-N6,N6-dimethyl-pyrido[3,4-d]pyrimidine4-4,6-diamine	15	C10	Tulobuterol hydrochloride	
07	C11	GW5074	3-(3, 5-Dibromo-4-hydroxybenzylidine-5-iodo-1,3-dihydro-indol-2-one)	15	C11	Tyrphostin 1	(4-Methoxybenzylidene)malonon itrile
07	D02	Edrophonium chloride	Ethyl(m-hydroxyphenyl)dimethylam monium chloride	15	D02	SKF 89976A hydrochloride	1-(4,4-Diphenyl-3-butenyl)-3-piperidinecarboxylic acid hydrochloride
07	D03	EGTA	Ethylene glycol-bis(2-aminoethylether)-N,N,N',N'-tetraacetic acid; Egtazic acid	15	D03	SR-95531	2-(3-Carboxypropyl)-3-amino-6-(4-methoxyphenyl)pyridazinium bromide
07	D04	Estrone	Folliculin	15	D04	SDZ-205,557 hydrochloride	4-Amino-5-chloro-2-methoxy-benzoic acid 2-(diethylamino)ethyl ester hydrochloride
07	D05	Alinidine	N-(2,6-Dichlorophenyl)-4,5-dihydro-N-2-propenyl-1H-imidazol-2-amine	15	D05	Tolbutamide	
07	D06	Furegrelate sodium	5-(3-Pyridinylmethyl)benzofuran carboxylic acid sodium	15	D06	Tetradecylthioacetic acid	TTA
07	D07	Foliosidine		15	D07	Trimipramine maleate	
07	D08	NS8593 hydrochloride	N-[(1R)-1,2,3,4-Tetrahydro-1-naphthalenyl]-1H-benzimidazol-2-amine hydrochloride	15	D08	N-p-Tosyl-L-phenylalanine chloromethyl ketone	TPCK
07	D09	Fluspirilene	R 6218	15	D09	Tyrphostin AG 555	Tyrphostin B46
07	D10	Guanfacine hydrochloride	N-(aminoiminomethyl)-2,6-dichloro-benzeneacetamide hydrochloride	15	D10	Trazodone hydrochloride	2-[3-[4-(3-Chlorophenyl)-1-piperazinyl]propyl]-1,2,4-triazolo[4,3-a]pyridin-3(2H)-one hydrochloride
07	D11	Genistein	5,7-Dihydroxy-3-(4-hydroxyphenyl)-4H-1-benzopyran-4-one	15	D11	Tyrphostin 23	3,4-(Dihydroxybenzylidene)malon onitrile
07	E02	Efaroxan hydrochloride	RX 821037A	15	E02	SIB 1757	6-Methyl-2-(phenylazo)-3-pyridinol
07	E03	(±)-Epinephrine hydrochloride	(±)-Adrenalin hydrochloride	15	E03	(±)-6-Chloro-PB hydrobromide	(±)-SKF-81297 hydrobromide
07	E04	Phenserine	(-)-N-Phenylcarbamoyleseroline	15	E04	SB 206553 hydrochloride	N-3-Pyridinyl-3,5-dihydro-5-methyl-benzo[1,2-b:4,5-b']dipyrrole-1(2H)-carboxamide hydrochloride
07	E05	Felbamate	2-Phenyl-1,3-propanediol dicarbamate	15	E05	Tetraethylthiuram disulfide	Disulfiram
07	E06	Fiduxosin hydrochloride	(3-[4-((3alphaR,9betaR)-cis-9-methoxy-1,2,3,3a,4,9b-hexahydro-[1]-benzopyrano[3,4-c]pyrrol-2-yl)butyl]-8-phenyl-pyrazino-[2',3':4,5]thi-eno [3,2-d]pyrimidine-2,4(1H,3H)-dione) hydrochloride	15	E06	Trequinsin hydrochloride	HL 725
07	E07	Fusaric acid	5-Butyl-2-pyridinecarboxylic acid	15	E07	Tyrphostin AG 490	2-propenamide

07	E08	Flunarizine dihydrochloride	1-[bis(4-fluorophenyl)methyl]-4-(3-phenyl-2-propenyl)-piperazine dihydrochloride	15	E08	(6R)-5,6,7,8-Tetrahydro-L-biopterin hydrochloride	
07	E09	cis-(Z)-Flupenthixol dihydrochloride	(Z)-4-[3-[2-(Trifluoromethyl)-9H-thioxanthen-9-ylidene]propyl]-1-piperazine-ethanol dihydrochloride	15	E09	Tyrphostin AG 698	Tyrphostin B52
07	E10	L-Glutamic acid hydrochloride	S(+)-1-Aminopropane-1,3-dicarboxylic acid hydrochloride	15	E10	Tyrphostin AG 34	Tyrphostin A24
07	E11	GW7647	2-(4-(2-(1-Cyclohexanebutyl)-3-cyclohexylureido)ethyl)phenylthio)-2-methylpropionic acid	15	E11	Pifithrin-mu	PFTmu; 2-Phenylethynesulfonamide
07	F02	Ellipticine	5,11-Dimethyl-6H-pyrido[4,3-b]carbazole	15	F02	SIB 1893	(E)-2-Methyl-6-[2-phenylethenyl]pyridine
07	F03	Ethosuximide	2-Ethyl-2-methylsuccinimide	15	F03	L-Beta-threo-benzyl-aspartate	(2S,3S)-2-Amino-3-benzylsuccinic acid
07	F04	N-Methyl-beta-carboline-3-carboxamide	FG-7142	15	F04	SB 224289 hydrochloride	1'-Methyl-5-([2'-methyl-4'-(5-methyl-1,2,4-oxadiazol-3-yl)biphenyl-4-yl] carbonyl)-2,3,6,7-tetrahydro-spiro(furo[2,3-f]indole-3,4'-piperidine)
07	F05	Fusidic acid sodium	Fusidin	15	F05	TCPOBOP	1,4-Bis-[2-(3,5-dichloropyridyloxy)]benzene
07	F06	Furosemide		15	F06	Tyrphostin AG 879	alpha-cyano-(3,5-di-t-butyl-4-hydroxy)thiocinnamide
07	F07	5-Fluorouracil	5-FU	15	F07	TTNPB	Arotinoid acid
07	F08	5-fluoro-5'-deoxyuridine	5'dFUrd	15	F08	Tyrphostin AG 527	Tyrphostin B44
07	F09	Furafylline	3-(2-Furanylmethyl)-3,7-dihydro-1,8-dimethyl-1H-purine-2,6-dione	15	F09	Tyrphostin AG 808	2-Cyano-3-(3',4'-dihydroxyphenyl)-1-(3"-indolyl)-3-oxo-1-propene
07	F10	Ganciclovir		15	F10	Triamcinolone	Fluoxyprednisolone
07	F11	alpha-Guanidinoglutaric acid	GGA	15	F11	Na-p-Tosyl-L-lysine chloromethyl ketone hydrochloride	TLCK hydrochloride
07	G02	Ebselen	2-Phenyl-1,2-benzisoselenazol-3(2H)-one	15	G02	1-(1-Naphthyl)piperazine hydrochloride	
07	G03	JX401	1-[2-Methoxy-4-(methylthio)benzoyl]-4-benzylpiperidine	15	G03	Suramin sodium salt	
07	G04	DPO-1	Diphenyl phosphine oxide-1	15	G04	L-Tryptophan	S(-)-1-alpha-Aminoindole-3-propionic acid
07	G05	Fenoterol hydrobromide	2-(3,5-Dihydroxyphenyl)-2-hydroxy-2'-(4-hydroxyphenyl)-1'-methyldiethylamine hydrobromide	15	G05	Tetraisopropyl pyrophosphoramide	iso-OMPA
07	G06	p-Fluoro-L-phenylalanine	4-Fluoro-L-phenylalanine	15	G06	Tetraethylammonium chloride	
07	G07	Flecainide acetate	N-(2-Piperidylmethyl)-2,5-bis-(2,2,2-trifluoroethoxy)benzamide acetate	15	G07	L-765,314	(2S)-4-(4-Amino-6,7-dimethoxy-2-quinazolinyl)-2-[[(1,1-Dimethylethyl)amino]carbonyl]-1-piperazinecarboxylic acid, phenylmethyl ester

07	G08	Flupirtine maleate	2-amino-6-[[(4-fluorophenyl)methyl]amino]-3-pyridinyl]-carbamic acid, ethyl ester maleate	15	G08	Theobromine	3,7-Dimethylxanthine
07	G09	FPL 64176	2,5-Dimethyl-4-[2-(phenylmethyl)benzoyl]-1H-pyrrole-3-carboxylic acid methyl ester	15	G09	Thio-NADP sodium	Thionicotinamide adenine dinucleotide phosphate sodium
07	G10	L-Glutamine	S(+)-Glutamic acid 5-amide	15	G10	S(-)-Timolol maleate	(S)-1-[(1,1-Dimethylethyl)amino]-3-[[4-(4-morpholinyl)-1,2,5-thiadiazol-3-yl]oxy]-2-propanol maleate
07	G11	Gallamine triethiodide		15	G11	Tyrphostin 25	(4,5-Trihydroxybenzylidene)malononitrile
07	H02	rac-2-Ethoxy-3-hexadecanamido-1-propylphosphocholine		15	H02	Ketanserin tartrate	R 41468
07	H03	Emodin		15	H03	SQ 22536	9-(Tetrahydro-2-furanyl)-9H-purin-6-amine
07	H04	(-)-Eseroline fumarate		15	H04	Tranilast	SB-252218
07	H05	S-(+)-Fluoxetine hydrochloride		15	H05	Tetramisole hydrochloride	(±)-2,3,5,6-Tetrahydro-6-phenylimidazo[2,1-b]thiazole hydrochloride
07	H06	Fluphenazine dihydrochloride		15	H06	Tolazamide	
07	H07	Fenoldopam bromide	SKF-82526	15	H07	Triamterene	
07	H08	Flutamide	2-Methyl-N-(4-nitro-3-[trifluoromethyl]phenyl)propanamide	15	H08	(±)-Taxifolin	Dihydroquercetin
07	H09	Fluoxetine hydrochloride	Prozac; LY-110,140 hydrochloride	15	H09	Tyrphostin AG 835	Tyrphostin B50
07	H10	Guanidinyl-naltrindole di-trifluoroacetate	GNTI di-trifluoroacetate	15	H10	N,N,N-trimethyl-1-(4-trans-stilbenoxy)-2-propylammonium iodide	F3
07	H11	GBR-12935 dihydrochloride	1-[2-(Diphenylmethoxy)ethyl]-4-(3-phenylpropyl)-piperazine dihydrochloride	15	H11	1-[2-(Trifluoromethyl)phenyl]imidazole	TRIM
08	A02	Isoguvacine hydrochloride	1,2,3,6-Tetrahydro-4-pyridinecarboxylic acid hydrochloride	16	A02	Taxol	Paclitaxel
08	A03	GYKI 52895	1-(4-Aminophenyl)-4-methyl-7,8-methylenedioxy-3,4-dihydro-5H-2,3-benzodiazepine hydrochloride	16	A03	Tomoxetine	(R)-N-methyl-gamma-(2-methylphenoxy)-benzenepropanamine
08	A04	MHPG piperazine	MOPEG piperazine	16	A04	Tamoxifen citrate	(Z)-2-[4-(1,2-Diphenyl-1-butenyl)phenoxy]-N,N-dimethyl-ethanamine cirtrate (1:1)
08	A05	DL-threo-beta-hydroxyaspartic acid	threo-2-Amino-3-hydroxysuccinic acid	16	A05	Telenzepine dihydrochloride	4,9-Dihydro-3-methyl-4-[(4-methyl-1-piperazinyl)acetyl]-10H-thieno[3,4-b][1,5]benzodiazepin-10-one dihydrochloride
08	A06	17alpha-hydroxyprogesterone	17alpha-Hydroxy-4-pregnene-3,20-dione	16	A06	Uridine 5'-diphosphate sodium	UDP

08	A07	L-Histidine hydrochloride	S(+)-alpha-Amino-1H-imidazole-4-propanoic acid hydrochloride	16	A07	U-69593	(+)-(5alpha,7alpha,8beta)-N-Methyl-N-[7-(1-pyrrolidinyl)-1-oxaspiro[4.5]dec-8-yl]-benzeneacetamide
08	A08	L-Hyoscyamine	[3(S)-endo]-alpha-(Hydroxymethyl)benzeneacetic acid 8-methyl-8-azabicyclo[3.2.1]oct-3-yl ester	16	A08	U-99194A maleate	5,6-Dimethoxy-2-(di-n-propylamino)indan maleate
08	A09	4-Hydroxybenzhydrazide	4-Hydroxybenzoylhydrazine	16	A09	Vincristine sulfate	VCR
08	A10	R-(+)-7-Hydroxy-DPAT hydrobromide	R(+)-7-Hydroxy-dipropylaminotetralin hydrobromide	16	A10	WIN 62,577	17-beta-Hydroxy-17-alpha-ethynyl-delta-4-androstano(3,2-b)pyrimido(1,2-a)benzimidazole
08	A11	Iodoacetamide		16	A11	Yohimbine hydrochloride	17-Hydroxyyohimban-16-carboxylic acid methyl ester hydrochloride
08	B02	Guvacine hydrochloride		16	B02	Tetracaine hydrochloride	
08	B03	GR-89696 fumarate	4-[(3,4-Dichlorophenyl)acetyl]-3-(1-pyrrolidinylmethyl)-1-piperazinecarboxylic acid methyl ester fumarate	16	B03	T-0156	2-(2-Methylpyridin-4-yl)methyl-4-(3,4,5-trimethoxyphenyl)-8-(pyrimidin-2-yl)methoxy-1,2-dihydro-1-oxo-2,7-naphthyridine-3-carboxylic acid methyl ester hydrochloride
08	B04	Hypotaurine	2-Aminoethanesulfinic acid	16	B04	Terfenadine	alpha-(4-[1,1-Dimethylethyl]phenyl)-4-[hydroxydiphenylmethyl]-1-piperidinebutanol
08	B05	Ciproxifan hydrochloride	Cyclopropyl[4-[3-(1H-imidazol-4-yl)propoxyl]phenyl]-methanone hydrochloride	16	B05	Thioperamide maleate	MR 12842
08	B06	1,3,5-tris(4-hydroxyphenyl)-4-propyl-1H-pyrazole	PPT	16	B06	U-74389G maleate	21-(4-[2,6-di-1-Pyrrolidinyl-4-pyrimidinyl]-1-piperazinyl)pregna-1,4,9[11]-triene-3,20-dione (Z)-2-butenedioate maleate
08	B07	(±)-8-Hydroxy-DPAT hydrobromide	(±)-8-Hydroxy-dipropylaminotetralin hydrobromide	16	B07	UK 14,304	5-Bromo-N-(4,5-dihydro-1H-imidazol-2-yl)-6-quinoxalinamine
08	B08	Hydroquinone	1,4-Benzenediol	16	B08	U0126	1,4-Diamino-2,3-dicyano-1,4-bis(o-aminophenylmercapto)butadiene
08	B09	Hemicholinium-3		16	B09	AMG 9810	2E-N-(2,3-Dihydro-1,4-benzodioxin-6-yl)-3-[4-(1,1-dimethylethyl)phenyl]-2-Propenamide
08	B10	GR 125487 sulfamate salt	[1-[2-[(Methylsulfonyl)amino]ethyl]-4-piperidinyl]methyl-5-fluoro-2-methoxy-1H-indole-3-carboxylate	16	B10	S(-)-Willardiine	S(-)-alpha-Amino-3,4-dihydro-2,4-dioxo-1(2H)-pyrimidinepropanoic acid
08	B11	HA-100	1-(5-Isoquinolinesulfonyl)piperazine hydrochloride	16	B11	YS-035 hydrochloride	N-[2-(3,4-Dimethoxyphenyl)ethyl]-3,4-dimethoxy-N-methyl-benzeneethanamine hydrochloride

08	C02	(±)-AMPA hydrobromide	(±)-alpha-Amino-3-hydroxy-5-methylisoxazole-4-propionic acid hydrobromide	16	C02	Tyrphostin 47	RG 50864
08	C03	Gabapentin	1-(Aminomethyl)-cyclohexaneacetic acid	16	C03	3-Tropanyl-3,5-dichlorobenzoate	MDL-72222
08	C04	Haloperidol		16	C04	Tropicamide	Ro 1-7683
08	C05	Hydroxytacrine maleate	HP-029	16	C05	(±)-Thalidomide	(±)-2-(2,6-Dioxo-3-piperidinyl)-1H-isoindole-1,3(2H)-dione
08	C06	1-(4-Hydroxybenzyl)imidazole-2-thiol		16	C06	Imiquimod	1-(2-Methylpropyl)-1H-imidazole[4,5-c]quinoline-4-amine
08	C07	Dopamine hydrochloride	3-Hydroxytyramine hydrochloride	16	C07	U-62066	Spiradoline mesylate
08	C08	BU99006	2-(Imidazolin-2-yl)-5-isothiocyanatobenzofuran	16	C08	Vinblastine sulfate salt	VLB
08	C09	HA-1004 hydrochloride	N-(2-Guanidinoethyl)-5-isoquinolinesulfonamide hydrochloride	16	C09	(±)-Vesamicol hydrochloride	AH-5183 hydrochloride
08	C10	IEM-1460	1-Trimethylammonio-5-(1-adamantanemethylammonio)pentane dibromide	16	C10	WAY-100635 maleate	N-[2-[4-(2-Methoxyphenyl)-1-piperazinyl]ethyl]-N-2-pyridinyl-cyclohexanecarboxamide maleate
08	C11	Ipratropium bromide	Atropine isopropyl bromide	16	C11	YC-1	3-(5'-Hydroxymethyl-2'-furyl)-1-benzyl indazole
08	D02	Muscimol hydrobromide	3-Hydroxy-5-aminomethylisoxazole hydrobromide	16	D02	Tyrphostin 51	2-Amino-1,1,3-tricyano-4-(3',4',5'-trihydroxyphenyl)butadiene
08	D03	DL-Homatropine hydrobromide	Tropine mandelate hydrobromide	16	D03	Trifluoperazine dihydrochloride	
08	D04	Hydralazine hydrochloride	1-Hydrazinophthalazine hydrochloride	16	D04	THIP hydrochloride	Gaboxadol hydrochloride
08	D05	Hydrocortisone	Cortisol	16	D05	R(+)-Terguride	R(+)-N,N-Diethyl-N'[(8alpha)-6-methylergolin-8-yl]urea
08	D06	Histamine dihydrochloride	1H-Imidazole-4-ethanamine dihydrochloride	16	D06	U-73122	1-[6-[[(17beta)-3-Methoxyestra-1,3,5(10)-trien-17-yl]amino]hexyl]-1H-pyrrole-2,5-dione
08	D07	Hydroxyurea		16	D07	S(-)-UH-301 hydrochloride	S(-)-5-Fluoro-8-hydroxy-DPAT hydrochloride
08	D08	MNS	3,4-Methylenedioxy-beta-nitrostyrene	16	D08	(±)-Verapamil hydrochloride	
08	D09	N-Methylhistaprodifen dioxalate salt	N-Methyl-2-[2-(3,3-diphenylpropyl)-1H-imidazol-4-yl]-ethanamine dioxalate salt	16	D09	XK469	2-(4-((7-Chloro-2-quinoxalinyl)oxy)phenoxy)propionic acid
08	D10	Ibudilast	KC-404	16	D10	AC-55649	4'-Octyl-4-biphenylcarboxylic acid, 4'-n-octylbiphenyl-4-carboxylic acid; 8BCA
08	D11	Idarubicin	Idamycin	16	D11	Zaprinast	1,4-Dihydro-5-(2-propoxyphenyl)-7H-1,2,3-triazolo[4,5-d]pyrimidin-7-one
08	E02	Guanabenz acetate	WY-8678	16	E02	PAC-1	(4-Benzylpiperazino)acetic acid (3-allyl-2-hyroxybenzylidene)hydrazide
08	E03	(±)-Vanillylmandelic acid	(±)-4-Hydroxy-3-methoxymandelic acid	16	E03	D-609 potassium	Carbonodithioic acid, O-(octahydro-4,7-methano-1H-inden-5-yl) ester potassium

08	E04	4-Imidazolemethanol hydrochloride	4-(Hydroxymethyl)imidazole hydrochloride	16	E04	Trifluperidol hydrochloride	R 2498 hydrochloride; Triperidol hydrochloride
08	E05	Lithium Chloride		16	E05	Thio-L-citrulline	N5-(Aminothioxomethyl)-L-ornithine
08	E06	Harmane	Aribine	16	E06	SKF 95282 dimaleate	N-[3-[3-(1-Piperidinylmethyl)phenoxy]propyl]-2-benzothiazolamine dimaleate; Zolantidine
08	E07	(+)-Hydrastine	(+)-beta-Hydrastine 1(S),9(R)	16	E07	R(+)-UH-301 hydrochloride	R(+)-5-Fluoro-8-hydroxy-DPAT hydrochloride
08	E08	Serotonin hydrochloride	5-HT hydrochloride	16	E08	VUF 5574	N-(2-methoxyphenyl)-N'-[2-(3-pyridinyl)-4-quinazolinyl]-urea
08	E09	Hexahydro-sila-difenidol hydrochloride, p-fluoro analog	p-F-HHSiD hydrochloride	16	E09	Wortmannin from Penicillium funiculosum	
08	E10	Imidazole-4-acetic acid hydrochloride	I4AA	16	E10	Xylazine hydrochloride	N-(2,6-Dimethylphenyl)-5,6-dihydro-4H-1,3-thiazin-2-amine hydrochloride
08	E11	Metolazone	7-Chloro-1,2,3,4-tetrahydro-2-methyl-3-(2-methylphenyl)-4-oxo-6-quinazolinesulfonamide	16	E11	Zonisamide sodium	1,2-Benzisoxazole-3-methanesulfonamide
08	F02	gamma-D-Glutamylaminomethylsulfonic acid	GAMS	16	F02	I-OMe-Tyrphostin AG 538	alpha-Cyano-(3-methoxy-4-hydroxy-5-iodocinnamoyl)-(3',4'-dihydroxyphenyl)ketone
08	F03	6-Hydroxymelatonin	3-(N-Acetylaminoethyl)-6-hydroxy-5-methoxyindole	16	F03	Thioridazine hydrochloride	
08	F04	Hexamethonium dichloride	N,N,N,N',N',N'-Hexamethyl-1,6-hexanediaminium dichloride	16	F04	3-Tropanyl-indole-3-carboxylate hydrochloride	ICS-205,930; Navoban; Tropisetron
08	F05	Hydrochlorothiazide	6-Chloro-3,4-dihydro-2H-1,2,4-benzothiadiazine-7-sulfonamide 1,1-dioxide	16	F05	Tyrphostin A9	[[3,5-bis(1,1-Dimethylethyl)-4-hydroxyphenyl]methylene]-propanedinitrile
08	F06	NG-Hydroxy-L-arginine acetate	NOHA acetate	16	F06	4-Imidazoleacrylic acid	Urocanic acid
08	F07	(±)-7-Hydroxy-DPAT hydrobromide	(±)-7-OH-DPAT HBr; (±)-Hydroxy-dipropylaminotetralin hydrobromide	16	F07	CGP 57380	N3-(4-fluorophenyl)-1h-pyrazolo[3,4-d]pyrimidine-3,4-diamine
08	F08	L-165,041	4-[3-(4-Acetyl-3-hydroxy-2-propylphenoxy)propoxy]phenoxyacetic acid	16	F08	Vinpocetine	Eburnamenine-14-carboxylic acid ethyl ester; (3alpha, 16alpha)-Eburnamenine-14-carboxylic acid ethyl ester
08	F09	Histamine, R(-)-alpha-methyl-, dihydrochloride	R(-)-alpha-Methylhistamine dihydrochloride	16	F09	1400W dihydrochloride	
08	F10	CPNQ	5-[4-(4-Chlorobenzoyl)-1-piperazinyl]-8-nitroquinoline	16	F10	Xamoterol hemifumarate	ICI 118587; (+/-)-N-[2-[[Hydroxy-3-(4-hydroxy)propyl]amino]ethyl-4-morpholinecarboxamide hemifumarate
08	F11	SB 228357	N-[3-Fluoro-5-(3-pyridinyl)phenyl]-2,3-dihydro-5-methoxy-6-(trifluoromethyl)-1H-indole-1-carboxamide	16	F11	Zardaverine	6-(4-Difluoromethoxy-3-methoxyphenyl)-3(2H)-pyridazinone
08	G02	Glipizide		16	G02	Tyrphostin AG 538	(alphaE)-alpha-[(3,4-Dihydroxyphenyl)methylene]-3,4-dihydroxy-beta-oxo-benzenepropanenitrile

08	G03	Hexamethonium bromide	Hexane-1,6-bis(trimethylammonium bromide)	16	G03	Thapsigargin	
08	G04	CCG-2046	3-Methyl-3-propyl-cyclopropane-1,1,2,2-tetracarbonitrile	16	G04	XCT790	-[4-(2,4-Bis-trifluoromethylbenzyloxy)-3-methoxyphenyl]-2-cyano-N-(5-trifluoromethyl-1,3,4-thiadiazol-2-yl)acrylamide
08	G05	SB 218795	(-)-(R)-N-(alpha-methoxycarbonylbenzyl)-2-phenylquinoline-4-carboxamide	16	G05	TPMPA	(1,2,5,6-Tetrahydropyridine-4-yl)methylphosphinic acid
08	G06	Retinoic acid p-hydroxyanilide	N-(4-Hydroxyphenyl)retinamide	16	G06	Urapidil hydrochloride	6[[3-[4-(o-Methoxyphenyl)-1-piperazinyl]propyl]amino]-1,3-dimethyluracil hydrochloride
08	G07	MHPG sulfate potassium	4-Hydroxy-3-methoxyphenylglycol-4-sulfate potassium	16	G07	(-)-trans-(1S,2S)-U-50488 hydrochloride	trans-(1S,2S)-3,4-Dichloro-N-methyl-N-(2-[1-pyrollidinyl]cyclohexyl)benzeneacetamide hydrochloride
08	G08	5-Hydroxy-L-tryptophan	S(+)-1-alpha-Amino-5-hydroxyindole-3-propionic acid	16	G08	Vancomycin hydrochloride from Streptomyces orientalis	Cancocin hydrochloride
08	G09	5-hydroxydecanoic acid sodium		16	G09	WB 64	N,N'-Tetramethyl-bis[(1,8-naphthylimid-9-yl)propyl-N,N'-hexane-1,6-diyl-bis-ammonium bromide
08	G10	NSC 95397	2,3-bis[(2-Hydroxyethyl)thio]-1,4-naphthoquinone	16	G10	Xylometazoline hydrochloride	2-(4-tert-Butyl-2,6-dimethylbenzyl)-2-imidazoline hydrochloride
08	G11	IMID-4F hydrochloride	2-[N-(2,6-dichlorophenyl)-N-(4-fluorobenzyl)amino]-2-imidazoline hydrochloride	16	G11	Olprinone hydrochloride	Loprinone hydrochloride; 1,2-Dihydro-5-(imidazo[1,2-a]pyridin-6-yl)-6-methyl-2-oxo-3-pyridinecarbonitrile hydrochloride
08	H02	GYKI 52466 hydrochloride	1-(4-Aminophenyl)-4-methyl-7,8-methylenedioxy-5H-2,3-benzodiazepine hydrochloride	16	H02	Trimethoprim	
08	H03	4-Hydroxy-3-methoxyphenylacetic acid	Homovanillic acid; HVA	16	H03	Tyrphostin AG 126	(3-Hydroxy-4-nitrobenzylidene)malononitrile
08	H04	6-Hydroxy-DL-DOPA	2,5-Dihydroxy-DL-tyrosine	16	H04	3-Tropanylindole-3-carboxylate methiodide	
08	H05	Hispidin	6-(3,4-dihydroxystyrl)-4-hydroxy-2-pyrone	16	H05	U-75302	6-[6-(3-Hydroxy-1E,5Z-undecadienyl)-2-pyridinyl]-1,5-hexanediol
08	H06	HE-NECA	2-Hexynyl-5'-ethylcarboxamidoadenosine	16	H06	Urapidil, 5-Methyl-	5-Methyl-6[[3-[4-(o-Methoxyphenyl)-1-piperazinyl]propyl]amino]-1,3-dimethyluracil
08	H07	5-Hydroxyindolacetic acid	5-HIAA	16	H07	U-101958 maleate	1-Benzyl-4-aminomethyl-N-[(3'-isopropoxy)-2'-pyridinyl]piperidine maleate
08	H08	Hydroxylamine hydrochloride		16	H08	(±)-gamma-Vinyl GABA	Vigabatrin
08	H09	R-(+)-8-Hydroxy-DPAT hydrobromide	(+)-8-Hydroxy-2-(dipropylamino)tetralin hydrobromide	16	H09	(R)-(+)-WIN 55,212-2 mesylate	(R)-(+)-[2,3-Dihydro-5-methyl-3[(morpholinyl)methyl]pyrrolo[1,2,3-de]-1,4-benzoxazinyl]-(1-naphthalenyl)methanone mesylate

08	H10	Imazodan	Cl 914	16	H10	Xanthine amine congener	8-[4-[[[[(2-Aminoethyl)amino]carbonyl] methyl]oxy]phenyl]-1,3-dipropylxanthine
08	H11	R(-)-Isoproterenol (+)-bitartrate		16	H11	Zimelidine dihydrochloride	(Z)-3-(4-Bromophenyl)-N,N-dimethyl-3-(3-pyridinyl)-2-propen-1-amine dihydrochloride

Supplementary Table S2

Primers used for SYBR-based real-time quantitative RT-PCR analyses.

Gene	Primers	Ann. Tmp.	Product size (bp)
Gapdh	Fw: 5'- TGCACCACCAACTGCTTAG -3'	60 °C	177
	Rv: 5'- GGATGCAGGGATGATGTTC -3'		
Runx2	Fw: 5'- CGGGCTACCTGCCATCAC -3'	60 °C	78
	Rv: 5'- GGCCAGAGGCAGAAGTCAGA -3'		
Osterix	Fw: 5'- CTCGTCTGACTGCCTGCCTAG -3'	60 °C	84
	Rv: 5'- GCGTGGATGCCTGCCTTGTA -3'		
Osteocalcin	Fw: 5'- CCGGGAGCAGTGTGAGCTTA -3'	60 °C	68
	Rv: 5'- AGGCGGTCTTCAAGCCATACT -3'		
Collagen 1a1	Fw: 5'- TGTCCCAACCCCCAAAGAC -3'	60 °C	92
	Rv: 5'- CCCTCGACTCCTACATCTTCTGA -3'		

Fw, forward; Rv, reverse
Ann. Temp.: Annealing temperature

Supplementary Figures

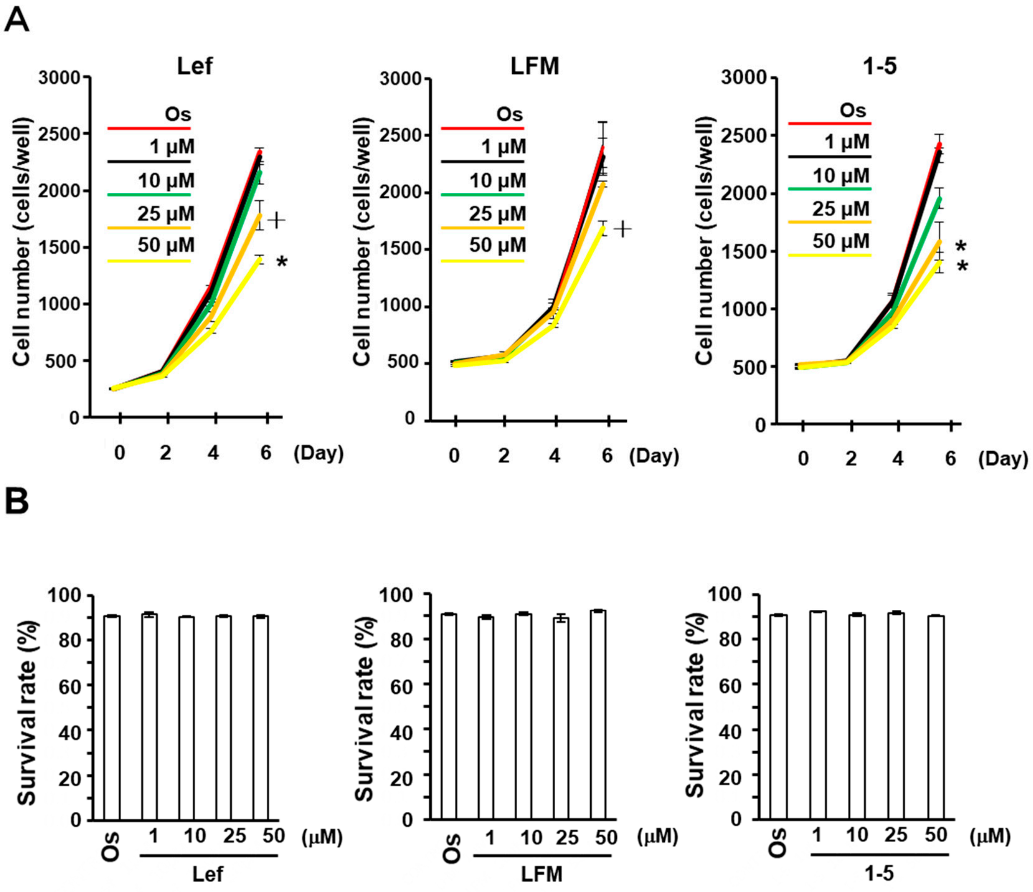

Supplementary Figure S1. Effects of identified candidate compounds on cytotoxicity and cell proliferation. **(A)** A cell proliferation assay was performed using MC3T3E1 cells in the presence or absence of leflunomide (Lef), LFM-A13 (LFM), and 1-(5-isoquinolinylsulfonil)-3-metylpiperazine dihydrochloride (1-5) (1–50 µM). The data represent the mean values ± standard deviation (n=10). Significant differences ($^+$ $P < 0.05$, * $P < 0.01$: ANOVA with Dunnett's correction for multiple comparisons) were evaluated in comparison to the control (osteogenic medium alone: Os) values at each time point. **(B)** The CytoTox-Glo luminescent cytotoxicity assay was performed using MC3T3E1 cells in the presence or absence of Lef, LFM, and 1-5 (1–50 µM). The number of dead and total cells was evaluated using a dead-cell protease assay and the survival rate of cells (cell viability) was calculated. The data represent the mean values ± standard deviation (n=3). Significant differences were not detected among the conditions (ANOVA with Dunnett's correction for multiple comparisons).

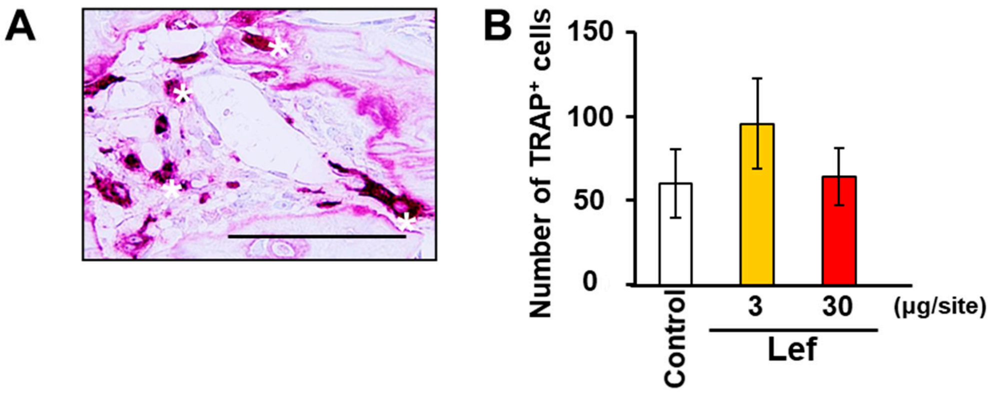

Supplementary Figure S2. Histological analysis for the effects of leflunomide (Lef) on osteoclast formation in the rat calvarial bone defect. After the defect (5 mm in diameter) had been formed, collagen graft material containing 0.5 or 5 μg of Lef was transplanted into the bone defects. Total 3 or 30 μg dosage of Lef was applied to each defect site dividing into 5 injections every three or four days. The same volume of saline was applied to the defect in the control group. At 3 weeks after the surgery, the tissues were collected and stained with tartrate-resistant acid phosphatase (TRAP) staining to count the number of $TRAP^+$ osteoclasts. **(A)** Histological image of multinucleated osteoclasts (*) stained with TRAP in the calvarial defects. Bars: 100 μm. **(B)** The number of osteoclasts in the bone defects for each condition was counted. The data represent the mean values ± standard deviation (n=3). Significant differences were not detected among the conditions (ANOVA with Dunnett's correction for multiple comparisons).
